# LncRNA *LncHrt* preserves cardiac metabolic homeostasis and heart function by modulating the LKB1-AMPK signaling pathway

**DOI:** 10.1007/s00395-021-00887-3

**Published:** 2021-08-11

**Authors:** Ning Liu, Masaharu Kataoka, Yingchao Wang, Linbin Pu, Xiaoxuan Dong, Xuyang Fu, Feng Zhang, Feng Gao, Tian Liang, Jianqiu Pei, Changchen Xiao, Qiongzi Qiu, Tingting Hong, Qiming Chen, Jing Zhao, Lianlian Zhu, Junhua He, Xiaoyun Hu, Yu Nie, Wei Zhu, Hong Yu, Douglas B. Cowan, Xinyang Hu, Jian’an Wang, Da-Zhi Wang, Jinghai Chen

**Affiliations:** 1 Department of Cardiology, Provincial Key Lab of Cardiovascular Research, Second Affiliated Hospital, Zhejiang University School of Medicine, Hangzhou, 310009 China; 2 Institute of Translational Medicine, Zhejiang University School of Medicine, Hangzhou, 310029 China; 3 Department of Cardiology, Boston Children’s Hospital, Harvard Medical School, 300 Longwood Avenue, Boston, MA 02115 USA; 4 Innovation Institute for Artificial Intelligence in Medicine of Zhejiang University, Hangzhou, 310018 China; 5 State Key Laboratory of Cardiovascular Disease, Fuwai Hospital, National Center for Cardiovascular Disease, Chinese Academy of Medical Sciences and Peking Union Medical College, Beijing, 100037 China; 6 Department of Gynecologic Oncology, Women’s Hospital, Zhejiang University School of Medicine, Hangzhou, 310029 China; 7 Harvard Stem Cell Institute, Harvard University, Cambridge, MA 02138 USA; 8 Present Address: Second Department of Internal Medicine, University of Occupational and Environmental Health, Kitakyushu, Japan

**Keywords:** LncRNA, Cardiac metabolic homeostasis, Cardiac remodeling, CDK5, SIRT2, LKB1-AMPK kinase cascade, Therapeutic target

## Abstract

Metabolic modulation is a promising therapeutic approach to prevent adverse remodeling of the ischemic heart. Because little is known about the involvement of long non-coding RNAs (lncRNAs) in regulating cardiac metabolism, we used unbiased transcriptome profiling in a mouse model of myocardial infarction (MI). We identified a novel cardiomyocyte-enriched lncRNA, called *LncHrt*, which regulates metabolism and the pathophysiological processes that lead to heart failure. AAV-based *LncHrt* overexpression protects the heart from MI as demonstrated by improved contractile function, preserved metabolic homeostasis, and attenuated maladaptive remodeling responses. RNA-pull down followed by mass spectrometry and RNA immunoprecipitation (RIP) identified SIRT2 as a *LncHrt*-interacting protein involved in cardiac metabolic regulation. Mechanistically, we established that *LncHrt* interacts with SIRT2 to preserve SIRT2 deacetylase activity by interfering with the CDK5 and SIRT2 interaction. This increases downstream LKB1-AMPK kinase signaling, which ameliorates functional and metabolic deficits. Importantly, we found the expression of the human homolog of mouse *LncHrt* was decreased in patients with dilated cardiomyopathy. Together, these studies identify *LncHrt* as a cardiac metabolic regulator that plays an essential role in preserving heart function by regulating downstream metabolic signaling pathways. Consequently, *LncHrt* is a potentially novel RNA-based therapeutic target for ischemic heart disease.

**Supplementary Information:**

The online version contains supplementary material available at 10.1007/s00395-021-00887-3.

## Introduction

Myocardial infarction (MI) is the leading cause of global mortality and morbidity [51] and typically occurs because of an acute blockage in a coronary artery. The ensuing tissue ischemia can cause cardiomyopathy, which is characterized by contractile dysfunction, scar formation, and structural remodeling [7, 74]. Cardiac remodeling responses to ischemia are initially functional, compensatory, and adaptive; but, when sustained, structural changes extend well beyond the region of injury, which leads to pathological hypertrophy accompanied by metabolic changes, including decreased fatty acid oxidation and increased glycolysis [19]. These effects decrease ATP synthesis, which, in turn, contributes to increased susceptibility of the injured heart to the progression toward failure [14, 19, 28, 45, 46]. Therefore, optimizing metabolic remodeling responses following myocardial infarction may provide an innovative therapeutic opportunity [19].

The mammalian heart is a ‘metabolic omnivore’ that prefers fatty acids as the fuel for oxidative phosphorylation. This preference can change based on environmental stress and is regulated at the level of enzyme activity as well as transcription, translation, and post-translational modification of proteins [22, 25]. During an ischemic episode, the activation of AMP-activated protein kinase (AMPK) is an adaptive response to cardiac energy deficit that prolongs the survival and function of cardiomyocytes. AMPK activation conserves energy by phosphorylating downstream targets that inhibit ATP-consuming anabolic processes and promote ATP-generating catabolic processes. By modulating cardiac metabolic homeostasis, AMPK activity decreases cardiomyocyte death and improves cardiac function [26, 52, 57]. Despite extensive study of AMPK and the upstream kinase LKB1 in regulating cardiac metabolic homeostasis, the role of long noncoding RNAs (lncRNAs) in these processes remain unclear.

Transcriptome analyses show thousands of lncRNAs are differentially expressed during heart development and disease [32, 47]. LncRNAs act as key modulators of gene expression that act epigenetically, post-transcriptionally, and translationally. Emerging evidence has shown that the manipulation of some lncRNAs can ameliorate dysfunction of the heart by regulating cardiac regeneration, hypertrophy, fibrosis, and angiogenesis [3, 43, 62, 64]. Our previous study showed that lincRNA-p21 plays a critical role in cell proliferation and neointimal formation during cardiovascular remodeling [68]. We also reported that many lncRNAs are dynamically expressed in ischemic cardiomyopathy patient samples, which results in extracellular matrix changes [20].

Using unbiased-screening of heart tissue 3 and 14 days after MI, we have previously reported that the expression of a cardiac fibroblast-enriched lncRNA, *Cfast*, was significantly induced in the heart of MI. Inhibition of *Cfast* preserved cardiac function by repressing fibrotic remodeling following cardiac injury [73]. Here, we report the identification of a cardiomyocyte-enriched lncRNA, named *LncHrt*, that is dramatically downregulated in infarcted hearts. We show *LncHrt* is essential for maintaining cardiac homeostasis and is sufficient to positively regulate cardiac metabolism and improve function post-MI. Mechanistically, *LncHrt* preserves cardiac metabolic balance through an interaction with SIRT2, which preserves its activity by interfering with the interaction between CDK5 and SIRT2. This abrogates the suppressive role CDK5 has on SIRT2 catalytic activity and activates downstream signaling of the LKB1-AMPK cascades to protect the heart from adverse remodeling responses.

## Materials and methods

### Animals

Experiments involving animals were performed in accordance with the Guide for the Care and Use of Laboratory Animals and were approved by the Institutional Animal Care and Use Committee (IACUC) of Boston Children’s Hospital (BCH) and by the IACUC of Zhejiang University. The animals were fed a standard laboratory diet and maintained with a 12:12 h light/dark cycle.

### Human tissue samples

Left ventricular (LV) and aorta tissues were taken from patients with dilated cardiomyopathy during heart transplantation. Surgeries were performed between September 2014 and February 2019. In brief, the patient’s heart was removed at the time of transplantation, LV and aorta tissues were dissected and snap frozen for subsequent analysis. LV samples from donated healthy hearts were used as a control. All subjects were duly informed and gave written consent. All studies were approved by the Ethics Review Committee of Second Affiliated Hospital of Zhejiang University.

### Microarray-based lncRNA profiling

Total RNAs were prepared from the heart tissues of mice 3 and 14 days after myocardial infarction (MI) (*n *= 5 hearts per group) or sham surgery (*n *= 4 hearts) (see below). The RNA samples were used for global lncRNA profiling with the LncRNA Microarray SurePrint G3 Gene Mouse GE 8 × 60K Kit (Agilent Technologies, Inc.) according to the manufacturer’s protocols. Agilent Feature Extraction software (version 11.0.1.1) was used to analyze acquired array images. Quantile-normalization and subsequent data processing were performed with the GeneSpring GX v12.1 software package (Agilent Technologies, Inc.). Differentially expressed LncRNAs were identified through filtering on Fold Change (≥ 2) and *P *value (*p* < 0.05).

### Quantitative reverse transcription-polymerase chain reaction (qRT-PCR)

Total RNA was isolated using TRIzol reagent (Life Technologies) from heart tissues and incubated with DNase I (Life technologies) to remove residual genomic DNA. For detecting RNA levels with qRT-PCR, one microgram RNA was used to synthesize cDNA following the manufacturer’s instructions (TAKARA). qRT-PCR signal was detected by the VII7 Real-time PCR System with a SYBR green qPCR Master Mix (Vazyme Biotech). ddCt was used to analyze gene expression from qPCR datasets. Data were normalized to 18S rRNA (*RN18s*) or Actin-beta (*Actb)* signal. Primers used for qRT-PCR in this study are listed in Supplementary Table 10.

### Isolation of adult mouse cardiac cells

Adult mouse cardiomyocyte and non-cardiomyocyte isolations were performed on 2- to 3-month-old mice using a previously reported procedure[5]. Briefly, the mouse heart was perfused and digested with collagenase II (Worthington Biochemical Corp, Lakewood, NJ), and dissociated cells were sedimented by gravity. The supernatant, enriched in non-cardiomyocytes, was collected by centrifuging for 5 min at 1000 rpm. The bottom layer, rich in cardiomyocytes, also was collected. The adult non-cardiomyocytes and cardiomyocytes were then treated with Trizol for RNA extraction and qRT-PCR analyses.

### RNA fractionation

Nuclear and cytoplasmic RNA fractions of cardiomyocytes were isolated using NE-PER Nuclear and Cytoplasmic Extraction Reagents (Thermo Scientific) according to the manufacturer’s instructions with addition of RNase inhibitor. The extracted nuclear and cytoplasmic RNA fractions were used to detect LncHrt subcellular content by qRT-PCR. The expression of *Actb* was used as a cytoplasmic control, and *U6* was used as a nuclear control.

### Rapid amplification of cDNA ends (RACE)

The full-length cDNA sequence of LncHrt was obtained from mouse heart RNA according to the SMARTer RACE 5ʹ/3ʹ Kit User Manual (TaKaRa). Nested 5ʹ and 3ʹ RACE products were obtained using GXL Taq polymerase with GC Buffer (Takara). The primers used for RACE PCR and nested PCR are presented in Supplementary Table 12. PCR products were extracted with a Gel Extraction kit (Vazyme Biotech), cloned into the pMD18-T vector, and analyzed by Sanger sequencing.

### AAV9 cloning, virus packaging and injection

HEK293T (TAKARA) cells were maintained in Dulbecco’s Modified Eagle’s Medium (DMEM, Sigma) supplemented with 10% fetal bovine serum (FBS; Gibco) and penicillin (100 U/mL)/streptomycin (100 µg/mL) (Life Technologies).

AAV9-cTNT:LncHrt (AAV-LncHrt) was generated in the AAV9 vector where the cardiac-specific TNNT2 promoter was used to drive the expression of LncHrt in the heart. An AAV9-cTNT:Luciferase (AAV-CTRL) was used as a control. The cDNA fragments encoding Luciferase and LncHrt were separately cloned into the AAV Inverted Terminal Repeat (ITR)-containing AAV9 plasmid (Penn Vector Core) harboring the chicken cardiac TNT promoter, to generate pEn.cTnT::Luciferase and pEn.cTnT::LncHrt, respectively. The AAV9-U6::LncHrt shRNA was generated using the pCRII Topo U6 vector, where the U6 promoter was used to drive the expression of the target LncHrt shRNA sequence in the heart to silencing LncHrt expression. The targeting sites of shRNA for LncHrt were designed using SirnaWizard (https://www.invivogen.com/sirnawizard/design.php), which are provide in Supplementary Table 7.

The AAV9 vectors were packaged in HEK293T cells with AAV9:Rep-Cap and pAd deltaF6 (Penn Vector Core) as described previously [15]. Transfections were performed using 1 mg/mL polyethylenimine, branched (Sigma) using a 1:4 dilution. After 60 h, cells were collected and lysed. AAV was purified and concentrated by gradient centrifugation. Titration of AAV particles was achieved using qRT- PCR, as described previously [12, 21]. The viral preparations had titers between 2 × 10^13^ and 5 × 10^13^ viral genome particles per mL. For AAV injection, 1-day-old neonatal C57BL/6 mice were randomly subjected to subcutaneous injection of AAV-LncHrt/AAV-LncHrt KD or AAV9-Control/Scramble (2 ~ 5 × 10^11^ viral genome particles per mouse heart), respectively; when mice were 2 months old, they were used to detect cardiac function or used for MI surgery. Intra-myocardial injections of AAV9-LncHrt or AAV9-Control (2 ~ 5 × 10^11^ viral genome particles per mouse heart), respectively, post-MI were performed as per our previous report [12]. Immediately after the ligation of LAD coronary artery, AAV9-LncHrt or AAV9-Control in a total volume of 30 μL was injected into ventricle muscular wall but not the ventricular cavity. The AAV9-LncHrt or AAV9-Control was evenly injected into four sites around the infarcted area (two sites in anterior wall, lateral wall, and apex area). Then, the chest was closed and the animal was placed in a prone position until recovery of spontaneous breathing.

### In vitro Cdk5 and Sirt2 silencing

Neonatal mouse cardiomyocytes (NMCMs) were isolated from 1- to 2-day-old C57BL/6 mice by enzymatic disassociation according to the manual of neonatal heart dissociation Kit (Miltenyi Biotec, Germany). Isolated cardiomyocytes were grown in M199 Medium containing 10% fetal bovine serum (FBS, Gibco) and Penicillin–Streptomycin (100 U/mL, Gibco) and 20 µM cytosine *β*-D-arabinofuranoside (Sigma). After 16–18 h, NMCMs were cultured in serum-free M199 for 24 h, then treated with siRNAs targeting Cdk5 (Gene Pharma Corp). Targeting siRNAs and scramble siRNAs were transfected into NMCMs at a final concentration of 50 nM using Lipofectamine RNAiMAX Reagent (Thermo). The NMCMs were also treated with 5 µM Sirt2 inhibitor sirReal2 (Selleck).

### Myocardial infarction

Myocardial infarction surgery was performed on 2-month-old C57BL/6 mice by ligation of the left anterior descending (LAD) coronary artery [60]. Mice were anesthetized with isoflurane. After the chest was shaved and cleaned with 75% alcohol, a suture was placed around the front upper incisors and pulled taut so that the neck was slightly extended. For oral intubation, the tongue was retracted and held with forceps, and a 20G catheter was inserted into the trachea. The catheter was then attached to the mouse ventilator via a Y-shaped connector. Ventilation was performed with a tidal volume of 225 µL for a 25-g mouse and a respiratory rate of 130 breaths per minute. 100% oxygen was provided to the inflow of the ventilator. Then the chest was opened through a left parasternal incision, and the heart exposed at the left 3rd–4th intercostal space. Chest retractor was applied to facilitate the view. The pericardium was opened, and the ligation was performed on the LAD coronary artery using 7–0 silk sutures (Ethicon). The lungs were slightly overinflated to assist in removal of air in the pleural cavity. Dissected intercostal space and chest skin were closed using a 6–0 silk suture (Ethicon). The sham group underwent the same surgical procedure (except that the LCA was not occluded).

### Measurement of cardiac function by echocardiography

Echocardiographic measurements were performed on mice using a Visual Sonics Vevo 2100 Imaging System (Visual Sonics, Toronto, Canada) with a 18–38 MHz transducer (model MS-400). Left ventricular (LV) dimensions, including diastolic and systolic wall thicknesses, LV end-diastolic and end-systolic chamber dimensions were measured from 2-D short-axis under M-mode tracings at the level of the papillary muscle. LV mass and functional parameters such as percentage of fractional shortening (FS%) and left ventricular volume were calculated using the above primary measurements and accompanying software.

Two-dimensional M-mode images of the left ventricle were captured at the level of the papillary muscles, Left ventricular (LV) dimensions, including diastolic (LVPW;d) and systolic (LVPW;s) wall thickness, and the left ventricular internal diameter at end-diastole (LVID;d) and end-systole (LVID;s) were measured. Percentage ejection fraction (EF) and percentage fractional shortening (FS) were calculated as described previously [70].

### Histology and immunostaining

Mouse hearts were dissected out, rinsed, and arrested in diastole buffer (4.7 nM KCl and 0.1% BDM in PBS). Hearts were then fixed in 4% paraformaldehyde (pH 7.4) overnight. After dehydration through a series of ethanol baths, samples were embedded in paraffin wax according to standard laboratory procedures. Sections of 4 µm in thickness were stained with hematoxylin and eosin (H&E). The stained sections were used for histological examination by light microscopy. To determine infarct size, the embedded paraffin blocks were cut through from apex to base. The first 5 sections of every 200 sections were used to stain with Fast Green and Sirius Red. Infarct size was calculated according to the following formula: [length of coronal infarct perimeter (epicardial + endocardial)/total left ventricular coronal perimeter (epicardial + endocardial)] × 100 [12, 50].

Immunofluorescence staining was performed on 4% paraformaldehyde-fixed and paraffin-embedded heart sections to measure the cardiomyocytes areas. After deparaffinization, rehydration, and heat-induced antigen retrieval, sections were incubated with wheat germ agglutinin (Alexa Fluor^®^ 647 conjugate WGA; 1:500, Invitrogen) for labeling the cardiomyocyte membrane. Image J software was used to measure the size of cardiomyocytes in the papillary muscles.

### RNA-seq and genome-wide transcriptome analysis

Total RNAs from the heart tissues of AAV-LncHrt and AAV-CTRL injected mice 7 days after MI was prepared for RNA-seq (three biological replicates for each group). RNA-seq experiments were performed by Novogene (Beijing, China). Briefly, total RNA was isolated from fresh ventricular tissue using TRIzol (Invitrogen). mRNA was then purified from total RNA using poly-T oligo-attached magnetic beads. Sequencing libraries were generated using NEBNext^®^ UltraTM RNA Library Prep Kit for Illumina^®^ (NEB, USA) following the manufacturer’s recommendations, and index codes were added to attribute sequences to each sample.

The clustering of the index-coded samples was performed on a cBot Cluster Generation System using TruSeq PE Cluster Kit v3-cBot-HS (Illuminia) according to the manufacturer’s instructions. After cluster generation, the library preparations were sequenced on an Illumina Hiseq platform and 150 bp paired-end reads were generated. For the data analysis, raw data (raw reads) in fastq format were first processed through in-house Perl scripts. Clean data (clean reads) were obtained by removing reads containing adapters, reads containing ploy-N, and low-quality reads from raw data. Reference genome and gene model annotation files were downloaded from genome website directly. Index of the reference genome was built using STAR and paired-end clean reads were aligned to the reference genome using STAR (v2.5.1b). STAR uses the method of Maximal Mappable Prefix (MMP). HTSeq v0.6.0 was used to count the read numbers mapped to each gene.

Analysis of differential expression was performed using the edgeR package (3.12.1). The resulting *P* values were adjusted using the Benjamini–Hochberg method for controlling the false discovery rate. Genes with an adjusted *P* value < 0.05 found by DESeq2 were assigned as differentially expressed. The hierarchical clustering heat map was generated with the ggplot library. Gene Ontology (GO) as well as Kyoto Encyclopedia of Genes and Genomes (KEGG) enrichment analysis of differentially expressed genes was implemented by the cluster Profiler R package, in which gene length bias was corrected. GO terms and KEGG pathways with corrected *P* value < 0.05 were considered significantly enriched in differential expressed genes.

### Mitochondrial respiration in permeabilized myocardial fibers

Approximately 10 mg intact myocardial tissue adjacent to border zone was dissected from each heart. The cardiac tissues were transferred into a petri dish with ice-cold BIOPS [35]. The tissues were separated mechanically into fiber bundles with two pairs of very sharp forceps, observing a change from red to pale coloring of the separated fiber bundles. Then the fiber bundles were permeabilized by gentle agitation for 30 min at 4 °C in ice cold BIOPS supplemented with 50 mg/ml saponin [61]. Myofibers were washed for 10 min by agitation in ice-cold mitochondrial respiration medium MiR05, blotted, weighed (Mettler Toledo microbalance XS205DU), and immediately used for respirometric measurements. Respiration was measured at 37 °C, with 2 mg permeabilized fibers in each high-resolution respirometry chamber containing 2 mL MiR05, using 2 chambers in parallel (OROBOROS Oxygraph-2k, Innsbruck, Austria). DatLab software (OROBOROS INSTRUMENTS) was used for data acquisition and analysis.

### ATP content detection

Myocardial ATP content detection was performed on fresh, infarcted heart tissues according to the manual of the Enhanced ATP Assay Kit (S0027; Beyonetime). Briefly, ATP was extracted by lysis buffer and measured by a luciferase reaction in which 560 nm light was emitted when D-luciferin was converted to oxyluciferin. Luminescence was measured using a luminometer (SpectraMax M5/M5e; Molecular Devices). ATP level was calculated according to a calibration curve with standard ATP samples. The total amount of tissue protein was used for normalization to ATP contents.

### Quantification of purine nucleotides

Myocardial purine nucleotides were extracted from frozen tissue samples with 0.6 M perchloric acid [1]. The extraction mixture was centrifuged for 10 min at 10,000 g and 4 °C, the supernatant was taken and quickly neutralized to pH = 6.5 ~ 7 with 1 M KOH solution. The neutralized supernatant was then allowed to stand for 5 min in an ice bath to precipitate the potassium perchlorate and then centrifuged for 10 min at 10,000 g and 4 °C. The neutralized supernatant was filtered through a 0.22-µm filter.

Purine nucleotides from the infarcted hearts were detected by HPLC (LC-20; SHIMADU) [18]. The purine nucleosides were separated on Ultimate AQ-C18 columns (250 × 4.6 mm). Peaks were detected and analyzed at 254 nm by a SPD-M20A diode array detector. Mobile phase A consisted of 60 mM dipotassium hydrogen phosphate and 40 mM potassium dihydrogen phosphate dissolved in deionized water, pH = 7, while mobile phase B consisted of 100% acetonitrile. HPLC separation was achieved using continuous gradient elution: 0 min 100% A, 0% B; 2 min 95% A, 5% B; 4 min 80% A, 20% B; 5.3 min 75% A, 25% B and 6 min 100% A, 0% B. Flow rate of the mobile phase was 1.2 mL/min, the injection volume was 20 µL. ATP, ADP and AMP in the samples were identified by comparison with retention time of standards, while the concentrations of ATP, ADP and AMP were determined according to the standard curve. The protein was dissolved in 50 mM NaOH at 4 °C overnight, and protein concentration was measured by BCA methods.

### Tagged-RNA pulldown

To identify the binding partners of LncHrt, we used a tagged-RNA pulldown assay, as described [37, 64]. Sense and antisense strands of streptavidin-binding S1m DNA were synthesized (Tsingke), annealed, and cloned into pCDH-MSCV at a cloning site, which was then used to insert sequences encoding LncHrt. The constructs expressing S1m, S1m-LncHrt as well as those expressing untagged LncHrt and EGFP were packaged into lentivirus using PEI (1:4) and then infected the cardiac fibroblasts with 20 MOI for 48 h. Cells were then harvested in SA-RNP lysis buffer (20 mM Tris–HCl (pH 7.5), 150 mM NaCl, 1.5 mM MgCl2, 2 mM DTT, 50 U/mL RNase OUT (Life Technologies) 50 U/mL Superase IN (Ambion) and 1 × complete protease inhibitor tablet (Roche). Streptavidin–Sepharose beads were blocked with 500 ng/µL yeast tRNA and 1 mg/mL BSA in SA-RNP lysis buffer before being added into cell lysates and being incubated at 37 °C for 2 h on a rotator. The beads were then pelleted and washed five times with SA-RNP washing buffer (20 mM Tris–HCl (pH 7.5), 300 mM NaCl, 5 mM MgCl2, 2 mM DTT, 50 U/mL RNase OUT (Life Technologies, NY, USA), 50 U/mL Superase IN (Ambion) and 1 × complete protease inhibitor tablet (Roche). After the last wash, RNA-bound proteins were then boiled in 50 µL 3 × LDS sample buffer (Life Technologies) and used for mass spectrometry.

### RNA immunoprecipitation (RIP)

RIP experiments were performed with the Magna RIPTM RNA-Binding Protein Immunoprecipitation Kit (Merck Millipore) as directed by the manufacturer. Briefly, 1-month-old mouse heart tissues were lysed thoroughly on ice in RIP Lysis Buffer supplemented with protease inhibitor cocktail and RNase inhibitor using a glass homogenizer to facilitate lysis. CDK5 and SIRT2 antibodies (Supplementary Table 8) with corresponding IgG were incubated with magnetic beads at room temperature for 1 h for each immunoprecipitation. After a 13,000 g centrifugation for 10 min, supernatants were incubated with the indicated antibody-bead complexes overnight at 4 °C with rotation. Co-immunoprecipitated RNAs were extracted as described by the kit manual, reverse-transcribed to cDNA, then subjected to qRT-PCR to measure LncHrt enrichment using primers listed in Supplementary Table 4. IgG enrichment served as negative controls.

### Immunoprecipitation (IP) and co-immunoprecipitation (Co-IP)

Heart tissues were lysed with immunoprecipitation (IP) buffer (Thermo) supplied with protease inhibitor cocktail (Roche) at 4 °C using a glass homogenizer to facilitate tissue lysis. Samples were then centrifuged at 13,000 g for 20 min, the lysates were precleared and 3 mg total protein/IP was incubated with the indicated primary antibodies overnight at 4 °C with rotation before incubation with Protein G Dynabeads (Invitrogen) for 6 h at 4 °C. The beads were then washed with high salt buffer once and IP buffer five times. 100 µL 3 × SDS loading buffer was added to the beads and boiled at 95 °C for 10 min prior to Western blot detection.

### Western blot analysis

Protein lysates were prepared from heart tissues in tissue extraction reagent (Invitrogen) supplemented with proteinase inhibitors. Cultured cells were harvested, homogenized, and incubated in Cell Extraction Buffer (Invitrogen) with protease inhibitor cocktail (Sigma) and 1 mM phenylmethyl sulfonyl fluoride (PMSF). Samples (50 µg total protein for each) were separated by 10% SDS–PAGE and electrophoretically transferred onto PVDF membranes. After blocking in 5% skimmed milk, membranes were incubated overnight at 4 °C with the indicated primary antibodies and then washed three times with TBST buffer before incubation for 1 h with HRP-conjugated secondary antibodies at room temperature. Protein bands were visualized using ECL Reagent (Invitrogen) with the Bio-Rad ChemiDoc imaging system. All the antibody information is listed in Supplementary Table 14.

### Statistical analysis

Statistical significance between two columns was assessed by two-tailed Student’s *t* test or Mann–Whitney test; for more than two columns, one-way ANOVA (Dunnett’s multiple comparisons test) or two-way ANOVA (Bonferroni's multiple comparisons test) analysis was used. Unless otherwise stated, results are presented as mean value ± standard error of the means (SEM).

## Results

### *LncHrt* is a cardiomyocyte-enriched lncRNA downregulated following myocardial infarction

To explore pivotal lncRNAs that regulate pathophysiological processes in heart disease, we performed microarray-based transcriptome profiling on mouse hearts 3 and 14 days after myocardial infarction (MI) and compared these to sham-operated control mice. An earlier report showed that *Cfast* is one of the most upregulated lncRNAs in MI hearts, and it regulates cardiac fibrotic remodeling following cardiac injury [73].

**Fig. 1:**
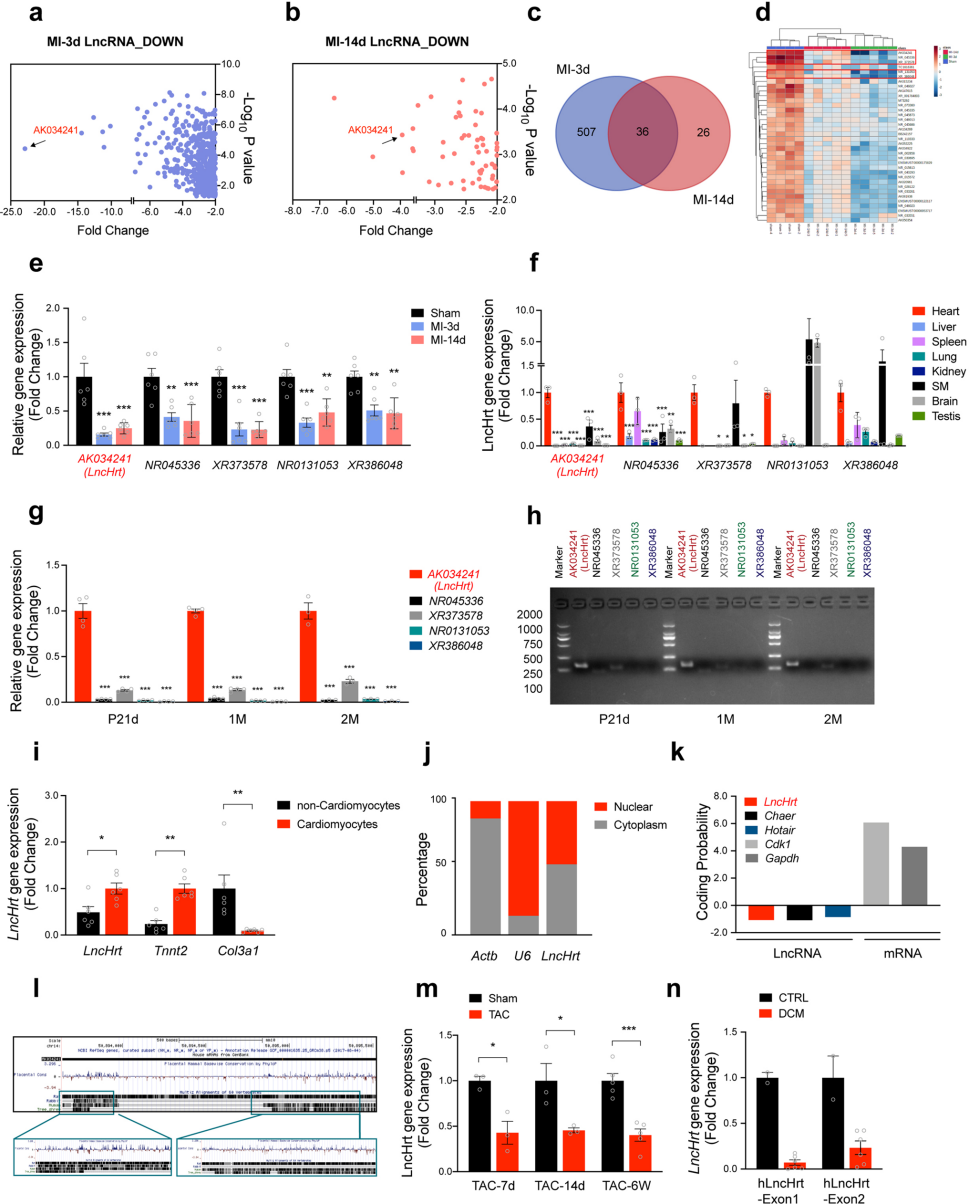
*LncHrt* is a cardiomyocyte-enriched lncRNA downregulated after myocardial infarction. **a** Volcano plot of downregulated lncRNAs in the heart tissues 3 days post-MI (MI-3d), Fold Change > 2, *P* value < 0.05. **b** Volcano plot of lncRNAs downregulated in the heart tissues 14 days post-MI (MI-14d), Fold Change > 2, *P* value < 0.05. **c** Venn plot of down regulated lncRNAs between MI-3d and MI-14d. **d** Heatmap of 36 downregulated lncRNAs in both MI-3d and MI-14d. **e** qRT-PCR of gene expression of 5 top downregulated lncRNAs (*AK034241, NR045336, XR373578, NR0131053, XR386048)* in the heart 3 days and 14 days after MI. *Rn18S* as a reference gene. Data are mean ± s.e.m. **P < 0.01 and ***P < 0.001 (versus Sham); by One-way ANOVA analysis, *n *= 6 mice per group. **f** Gene expression of 5 top downregulated lncRNAs in different mouse tissues by qRT-PCR. *Rn18S* as a reference gene. Data are mean ± s.e.m. *P < 0.05, **P < 0.01 and ***P < 0.001 (versus Heart); by One-way ANOVA analysis, *n *= 3 mice per group. **g** Gene expression of 5 top downregulated lncRNAs in heart tissues of postnatal 21 days (P21d), 1 month (1 M) and 2 months (2 M) old mice. *Rn18S* as a reference gene. Data are mean ± s.e.m. *P < 0.05, **P < 0.01 and ***P < 0.001 (versus *LncHrt*); by One-way ANOVA analysis, *n *= 4 mice per group. **h** Image of agarose electrophoresis showing RT-PCR of *AK034241, NR045336, XR373578, NR0131053, XR386048* in P21d, 1 M and 2 M old heart tissues. **i** *LncHrt* expression in cardiomyocytes and non-cardiomyocytes. *Rn18S* as a reference gene. Data are mean ± s.e.m. *P < 0.05 and **P < 0.01 (versus Cardiomyocytes); by Student’s *t* test, *n *= 6 mice per group. **j** Subcellular location of *LncHrt* in cardiomyocytes. *Actb* acts as a cytoplasm marker while *U6* as a nuclear marker. **k** Coding probability of *LncHrt* predicted by Coding Potential Calculator (CPC). Protein coding mRNAs *Cdk1* and *Gapdh* serve as negative controls and reported LncRNAs *Chaer* and *Hotair* are positive controls. **l** Conservation analysis of *LncHrt* in different mammalian species in UCSC Genome Browser on Mouse Dec. 2011 (GRCm38/mm10) Assembly. **m** qRT-PCR detection of *LncHrt* gene expression in mice TAC model. *Rn18S* as a reference gene. *n *= 3 or 5 per group. **n** qRT-PCR detection of the human homology *LncHrt* gene expression in human hearts with dilated cardiomyopathy (DCM). *Rn18S* as a reference gene. *n *= 2 or 6 human heart samples per group

The present study focused on downregulated lncRNAs. Transcriptome analysis revealed 543 lncRNAs downregulated (fold change > 2, *P* < 0.05) in mouse hearts 3 days post-MI (MI-3d, Fig. 1a), and 62 lncRNAs downregulated (fold change > 2, *P* < 0.05) in hearts 14 days post-MI (MI-14d, Fig. 1b). Using combined analyses, we identified a total of 36 lncRNAs downregulated in both MI-3d and MI-14d time points (Fig. 1c, d and Supplementary Table1). Among them, the top five most downregulated lncRNAs are *AK034241, NR_045336, XR_373578, NR_131053, and XR_386048*. In particular, the expression of *AK034241* decreased over 22-fold (Fig. 1a–d and Supplementary Table 1).

We validated these top five downregulated lncRNAs using an independent batch of MI heart tissues by quantitative reverse transcription polymerase chain reaction (qRT-PCR) (Fig. 1e). To determine the tissue distribution of these lncRNAs, we assessed their expression in different tissues. We found that *AK034241* and *XR_373578* was specifically enriched in the heart (Fig. 1f), indicating their potential relevance in cardiac disease. Intriguingly, *AK034241* was the most abundantly expressed lncRNA in heart tissue compared to the other 4 lncRNAs (Fig. 1g, h). As a result, we focused on *AK034241* and named it *lncRNA-heart* (*LncHrt)*.

Next, we investigated the developmental expression pattern of *LncHrt* (*i.e.,* from neonates to adulthood) and found that *LncHrt* was progressively elevated 7 days after birth and then dramatically increased until adulthood, indicating *LncHrt* may be essential for adult heart homeostasis. (Fig. S1, a, b). To determine the cell type expressing *LncHrt*, we isolated cardiomyocytes and non-cardiomyocytes from adult mouse hearts and found *LncHrt* was primarily expressed in adult cardiomyocytes (Fig. 1i). In addition, we performed fractionation assays to determine the subcellular location of *LncHrt*. qRT-PCR assays detected that *LncHrt* was equally distributed in the nucleus and cytoplasm of cardiomyocytes (Fig. 1j), suggesting that *LncHrt* may participate in both transcriptional and post-transcriptional regulatory processes.

We performed 5ʹ and 3ʹ rapid amplification of cDNA ends (RACE) using RNA isolated from adult mouse hearts and demonstrated the full length of *LncHrt* is 1907 nt (Fig. S1, c, d). There were an additional 9 nt in the 5ʹ end compared to annotated *AK034241*. The *LncHrt* gene contains two exons, and like many lncRNAs, it has a poly(A) tail. We evaluated if the *LncHrt* transcript is a true non-coding RNA using the Coding Potential Calculator [30] and Coding Potential Assessment Tool [63]. As expected, both revealed that the *LncHrt* transcript has a very low coding-potential score, similar to *Chaer* [64] and *Hotair* [13], two well-known lncRNAs, and unlike protein-coding genes, like *Cdk1* and *Gapdh*, which have high coding-potential scores (Fig. 1k and Fig. S1e).

We subsequently examined the conservation of *LncHrt* using the UCSC Genome Browser (GRCm38/mm10). The genome locus of *LncHrt* in humans is conserved, while the *LncHrt* sequence is highly conserved across multiple species in exon 1 as well as the 5ʹ-end and 3ʹ-end of exon 2 (Fig. 1l). The predicted *LncHrt* secondary structure based on minimum free energy (MFE) and partition function was assessed using the RegRNA Server (http://rna.tbi.univie.ac.at/cgi-bin/RNAWebSuite/RNAfold.cgi). These analyses revealed that *LncHrt* has stem-loop structures with relatively high base-pairing potential (Figure. S1f).

Consistent with the finding that mouse *LncHrt* was downregulated in the heart after myocardial infarction, we also found that *LncHrt* was decreased in the murine transverse aortic constriction (TAC) model and the expression of the human homolog of mouse *LncHrt* was reduced in patients with dilated cardiomyopathy (DCM) (Fig. 1m, n and Supplementary Table 2). These findings suggest that this lncRNA may play an important role in the regulation of heart disease.

### Knock-down of *LncHrt* impairs cardiac homeostasis

**Fig. 2:**
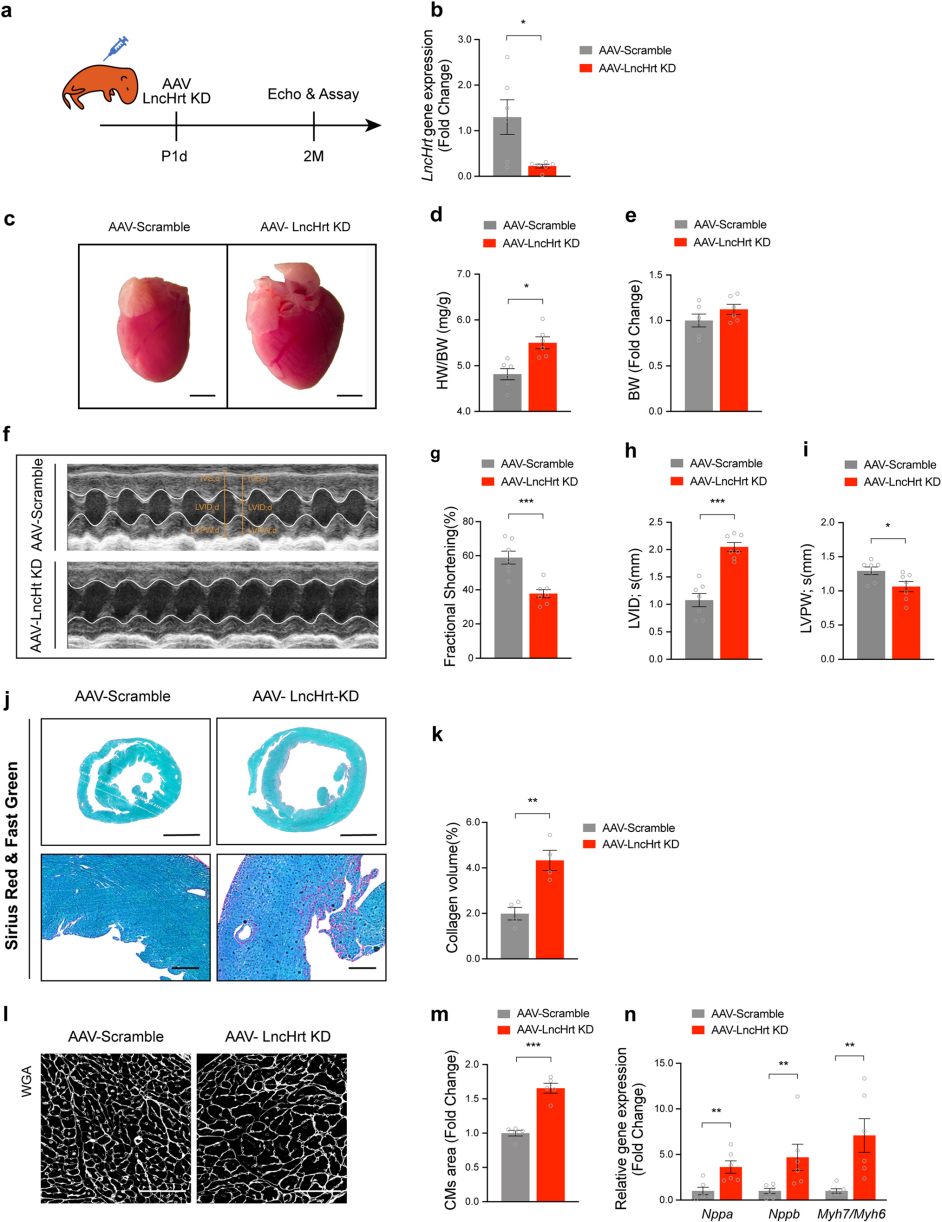
Knock-down of *LncHrt* impairs cardiac homeostasis. **a** Schematic diagram showing the experimental procedures for AAV9 delivered LncHrt knocking down (KD) in mice. **b** qRT-PCR of *LncHrt* gene expression. *Rn18S* as a reference gene. Data are mean ± s.e.m. *P < 0.05 and **P < 0.01 (versus AAV-Scramble); by Mann–Whitney test, *n *= 6 mice per group. **c** Representative images of gross morphology of hearts from AAV-Scramble and AAV-LncHrt KD. Scale bar = 2 mm. **d** Ratio of heart weight (HW) to body weight (BW) in AAV-Scramble and AAV-LncHrt KD heart at 2–3 months. Data are mean ± s.e.m. *P < 0.05 and **P < 0.01 (versus AAV-Scramble); by Student’s* t *test, *n *= 6 mouse per group. **e** Body weight (BW) in AAV-Scramble and AAV-LncHrt KD heart at 2–3 months. Data are mean ± s.e.m. *P < 0.05 and **P < 0.01 (versus AAV-Scramble); by Student’s* t *test, *n *= 6 mice per group. **f** M-mode echocardiography of mice heart 2–3 months after AAV9 delivered LncHrt-shRNA injection. **g**-**i** Echocardiography analyses of cardiac function of FS% (**g**), LVID (**h**) and. LVPW (**i**) after AAV9 delivered LncHrt-shRNA 2–3 months compared to their scramble group. FS, left ventricular fractional shortening. LVID,s, LV internal dimension at end-systole. LVPWs, left ventricular posterior wall at end-systole. Data are mean ± s.e.m. *P < 0.05 and **P < 0.01 (AAV-Scramble); by Student’s* t *test, *n *= 7 mice per group. **j** Representative of low- (top) and high-magnification (bottom) of images of Sirius red & fast green staining of scramble and LncHrt knock-down heart sections, Scale bars, 2 mm (up), 50 μM (down). **k** Quantification of collagen volume of scramble and LncHrt knocking down hearts. Data are mean ± s.e.m. *P < 0.05 and **P < 0.01 (versus AAV-Scramble); by Student’s* t *test, *n *= 4 mice. **l** Representative pictures of cardiomyocytes stained with WGA. Scale bars = 100 µm. **m** Quantification of cardiomyocyte area of AAV-LncHrt KD compared to AAV-Scramble group. Data are mean ± s.e.m. ***P < 0.001 (versus AAV-Scramble); by Student’s* t *test, *n *= 5 mice hearts per group, measured ~ 400 cardiomyocytes per heart and calculated median value for each heart. **n** Gene expression of cardiac disease markers in scramble and LncHrt knock down hearts. *Rn18S* as a reference gene. Data are mean ± s.e.m. *P < 0.05 and **P < 0.01 (versus AAV-Scramble); by Student’s* t *test (*Nppa*) or Mann–Whitney test (*Nppb*, *Myh7*/*Myh6*), *n *= 6 mice per group

To define the in vivo function of *LncHrt* in the heart, we knocked down (KD) *LncHrt* expression using AAV9-shRNA (short hairpin RNA) directed against *LncHrt* (AAV-LncHrt KD) in neonatal mice, as previously reported [10]. For the control group, the mice received an AAV9 expressing a scrambled short hairpin RNA sequence (AAV-Scramble) (Fig. 2a). AAV-LncHrt KD resulted in a decreased cardiac expression of *LncHrt* by 75% compared to AAV-Scramble (Fig. 2b) and the hearts of AAV-LncHrt KD mice were enlarged compared to control mice (Fig. 2c). The heart weight to body weight ratio (HW/BW) was also significantly increased in these mice (Fig. 2d) with no effect on mice body weight (Fig. 2e).

Echocardiography revealed that AAV-LncHrt KD impaired left ventricular systolic heart function (Fig. 2f and Supplementary Table 3) and decreased fractional shortening (Fig. 2g). In particular, AAV-LncHrt KD hearts exhibited a dilated left ventricular chamber [LVID,s (mm), Fig. 2h and Supplementary Table 3] and a reduced left ventricular posterior wall thickness [LVPW,s (mm), Fig. 2i and Supplementary Table 3].

We validated these structural changes histologically and assessed fibrosis in AAV-Scramble and AAV-LncHrt KD treated hearts (Fig. 2j, k). These hearts were also used to assess cardiomyocyte size (Fig. 2l and m). Marker analyses demonstrated a significant increase in the expression of cardiomyopathy marker genes such as *Nppa*, *Nppb,* and *Mhy7/Myh6* after *LncHrt* knockdown (Fig. 2n), which supports our observation that knock-down of *LncHrt* adversely affects cardiac function. To confirm this, and rule out the possibility of off-target effects resulting from shRNA-mediated *LncHrt* knock-down, we designed another *LncHrt shRNA to* target a different site of the *LncHrt* gene (AAV-LncHrt KD-A) and obtained similar results (*i.e.,* impaired heart function and increased pathological remodeling) (Fig. S2, a–n and Supplementary Table 3). Together, these data indicate that *LncHrt* plays an essential role in maintaining normal cardiac function.

### *LncHrt* overexpression protects the heart from myocardial infarction

**Fig. 3:**
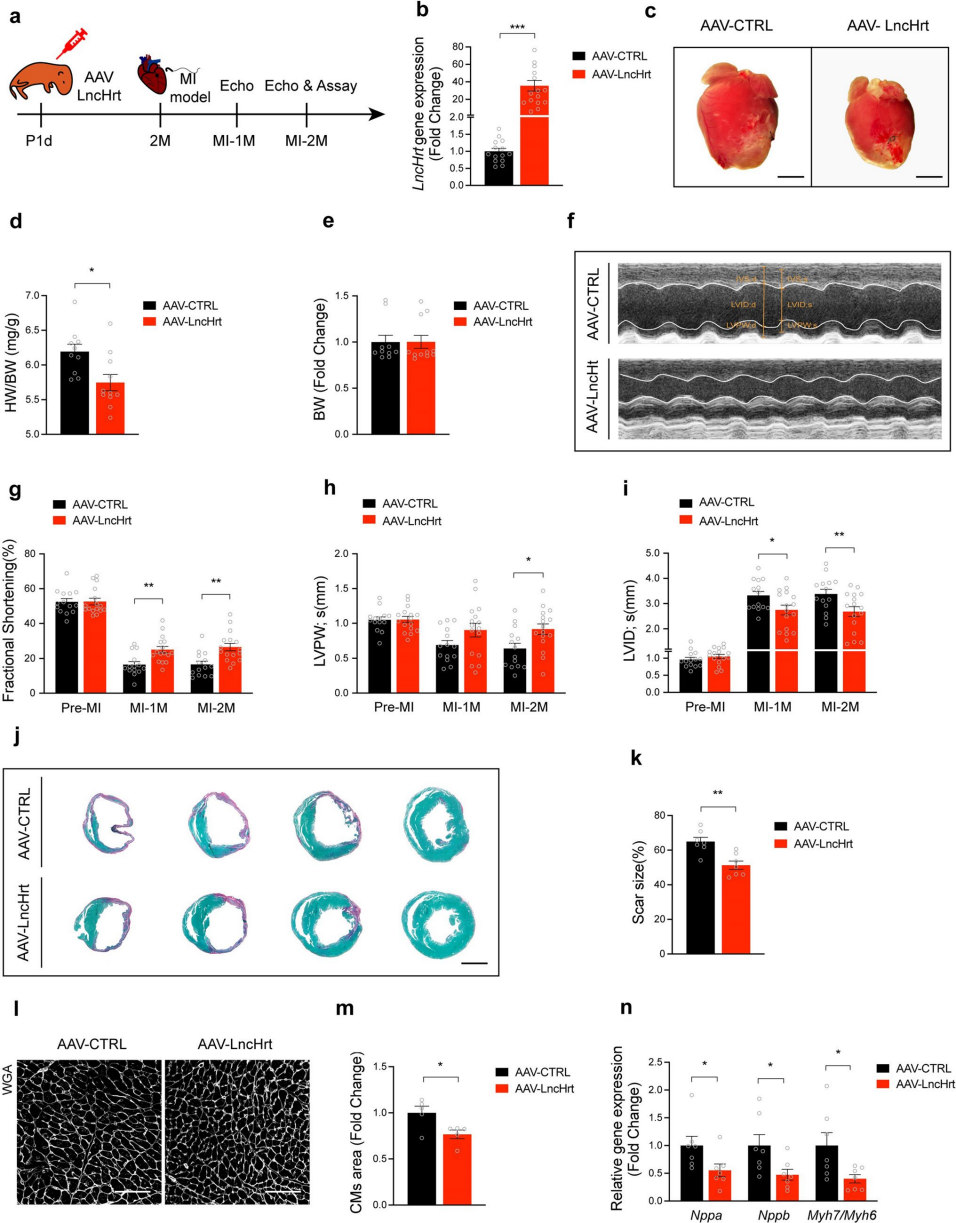
AAV9-delivered *LncHrt* overexpression protects the heart from myocardial infarction. **a** Schematic diagram showing the experimental design with AAV9-delivered *LncHrt* overexpression in mice hearts with myocardial infarction injury. Echocardiography was performed at Pre-MI, post-MI 1 month (MI-1M) and 2 months (MI-2M). **b** qRT-PCR of *LncHrt* gene expression after MI-2M. *Rn18S* as a reference gene. Data are mean ± s.e.m. *P < 0.05, **P < 0.01, ***P < 0.001 (versus AAV-CTRL); by Mann–Whitney test, *n *= 14–16 mice per group. **c** Representative images of gross morphology of hearts from AAV-CTRL and AAV-LncHrt. Scale bar = 2 mm. **d** Ratio of heart weight (HW) to body weight (BW) in AAV-CTRL and AAV-LncHrt heart post-MI (2 months). Data are mean ± s.e.m. *P < 0.05 and **P < 0.01 (versus AAV-CTRL); by Student’s* t *test, *n *= 10–11 mouse per group. **e** Body weight (BW) in AAV-CTRL and AAV-LncHrt heart post-MI (2 months). Data are mean ± s.e.m. *P < 0.05 and **P < 0.01 (versus AAV-CTRL); by Student’s* t *test, *n *= 10–11 mice per group. **f** M-mode echocardiography of mice heart 2 months after AAV9 delivered LncHrt injection. **g**-**i** Analyses of cardiac function of FS% (g), LVPW (h) and LVID (i) after AAV-LncHrt injection at pre-MI, 1 and 2 months post-MI compared to their CTRL group. FS, left ventricular fractional shortening. LVPWs, left ventricular posterior wall at end-systole. LVIDs, LV internal dimension at end-systole. Data are mean ± s.e.m. *P < 0.05 and **P < 0.01 (versus AAV-CTRL); by 2-way ANOVA analysis, *n *= 14–16 mice per group. **j** Representative of images of Sirius red/fast green collagen staining of series of transverse sections after injection of AAV-LncHrt compared to AAV-CTRL group after MI injury. Sirius red/fast green collagen staining marks myocardium (green) and scar (red). Scale bars = 2 mm. **k** Quantification of scar size of AAV-LncHrt compared to AAV-CTRL group after MI injury. Data are mean ± s.e.m. *P < 0.05 and **P < 0.01 (versus AAV-CTRL); by Student’s* t *test, *n *= 7 mice per group. **l** Representative images of cardiomyocytes stained with WGA. Scale bars = 100 μm. **m** Quantification of cardiomyocytes area of AAV-LncHrt compared to AAV-CTRL group after MI injury. Data are mean ± s.e.m. *P < 0.05, **P < 0.01 and ***P < 0.001 (versus AAV-CTRL); by Student’s* t *test, *n *= 5 mouse hearts per group, measured ~ 300 cardiomyocytes per heart and calculated median value for each heart. **n** Hypertrophic marker gene expression of heart of AAV-LncHrt versus AAV-CTRL group. *Rn18S* as a reference gene. Data are mean ± s.e.m. *P < 0.05 and **P < 0.01 (versus AAV-CTRL); by Student’s* t *test or Mann–Whitney test, *n *= 7 mice per group

Because inhibition of *LncHrt* expression negatively impacts cardiac function, and decreased *LncHrt* levels were found in the hearts after MI, we tested the hypothesis that overexpression of *LncHrt* could play a protective role in the pathological progression of MI. We employed AAV9-mediated delivery system to overexpress *LncHrt *in vivo using the cTNT promoter to achieve cardiomyocyte-specific overexpression of *LncHrt* (AAV-LncHrt). An AAV9-luciferase construct was used as a control (AAV-CTRL) (Fig. 3a). qRT-PCR analysis confirmed increased *LncHrt* cardiac expression 2 months after AAV-LncHrt treatment (Fig. 3b). Triphenyltetrazolium chloride (TTC) viability staining validated that MI induced infarct size is comparable between the two groups (Fig. S3, a, b). Overexpression of *LncHrt* improved cardiac morphology and significantly reduced the HW/BW ratio in response to MI compared to the control group (Fig. 3c, d) without affecting mice body weight (Fig. 3e). More importantly, overexpression of *LncHrt* ameliorated cardiac function after MI (Fig. 3f), as determined by echocardiography that showed improved fractional shortening (FS%) (Fig. 3g and Supplementary Table 4), maintenance of left ventricular posterior wall thickness [LVPW,s (mm), Fig. 3h and Supplementary Table 4], and reduced cardiac chamber internal diameter [LVID,s (mm), Fig. 3i and Supplementary Table 4].

Histological analyses revealed that overexpression of *LncHrt* in the heart substantially reduced infarct fibrosis following MI when compared to controls (Fig. 3j, k). In addition, cardiomyocyte size was markedly decreased in AAV-LncHrt treated hearts post-MI (Fig. 3l, m), indicating reduced cardiac pathological remodeling. Molecular marker analyses demonstrated a significant decrease in the expression of the hypertrophic and cardiomyopathy marker genes *Nppa, Nppb,* and *Myh7/Myh6* in AAV-LncHrt-treated hearts (Fig. 3n). We also assessed the effects of *LncHrt* on healthy mouse hearts as a baseline. We found that *LncHrt* did not affect cardiac function and heart morphology in healthy hearts (Fig. S4, a–j and Supplementary Table 5). Together, these results indicate that *LncHrt* plays a role in preserving cardiac function and attenuates adverse remodeling in response to MI.

### *LncHrt* overexpression rescues the transcriptome in infarcted hearts

**Fig. 4:**
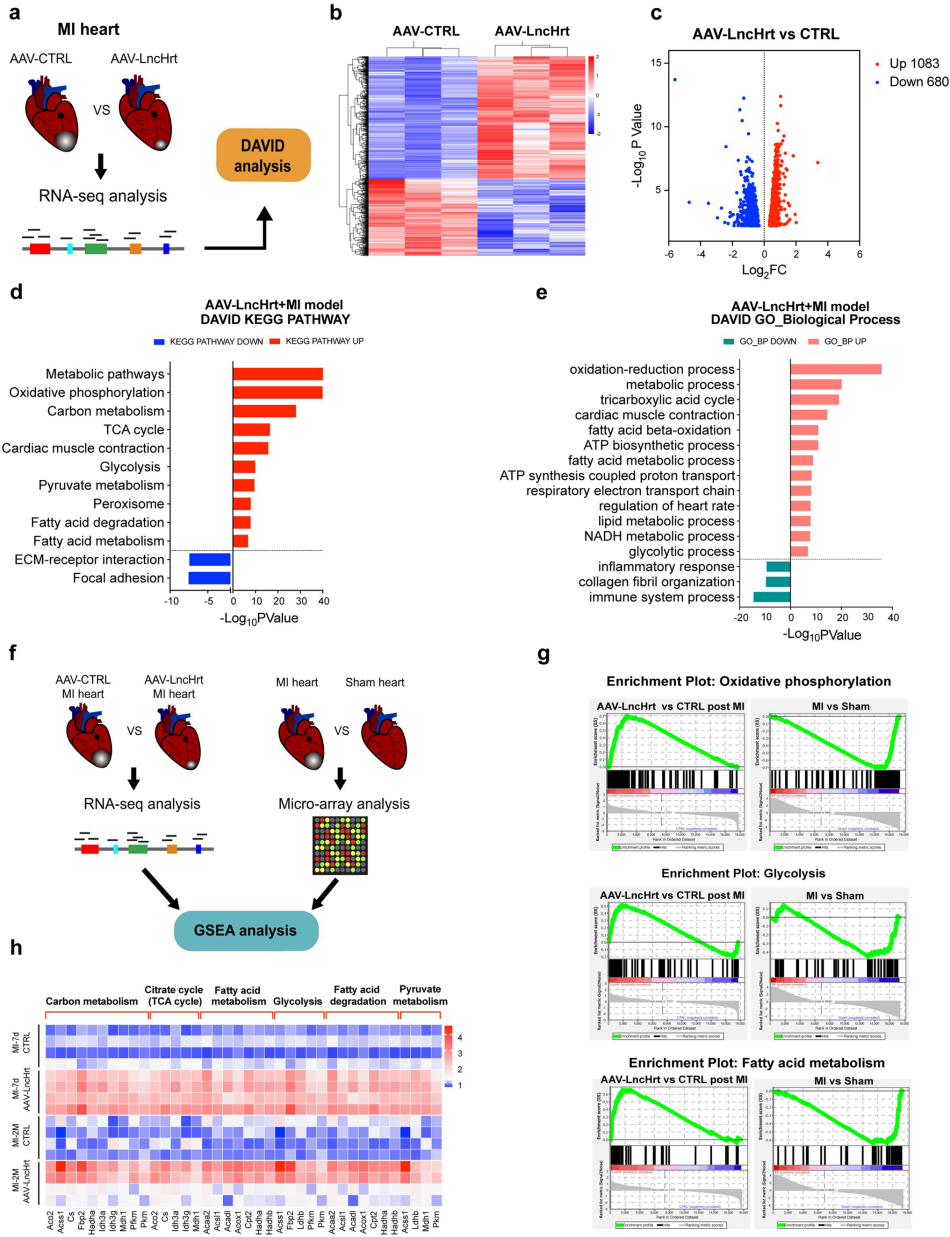
Cardiac *LncHrt* overexpression rescued transcriptomes of myocardial infarction. **a** Schematic diagram showing the experimental design of RNA-sequencing (seq) in AAV-LncHrt vs. CTRL post-MI, *n *= 3 mice per group. **b** Hierarchical clustering of differentially expressed genes in AAV-LncHrt vs. CTRL hearts as assessed by RNA-seq (adjusted *P* value < 0.05). **c** Volcano plot of differentially expressed genes in AAV-LncHrt vs. CTRL hearts (Log_2_Fold Change > 0.55, adjusted *P* Value < 0.05). **d** KEGG pathway enrichment analysis of differentially expressed genes in AAV-LncHrt vs. CTRL hearts post myocardial infarction using DAVID tools. **e** Gene Ontology (GO) analysis linked to biological process of differentially expressed genes in AAV-LncHrt vs. CTRL hearts post myocardial infarction using DAVID tools. **f** Schematic diagram showing the experimental design of combined analysis of AAV-LncHrt RNA-seq post-MI and Micro-array of MI heart by Gene set enrichment analysis (GSEA). **g** GSEA analysis of metabolic related enrichment plots which inhibited in MI were rescued by *LncHrt*. **h** Heat maps of metabolic related genes validation by qRT-PCR using an independent batch of samples post-MI-7d and MI-2 M

To gain a full understanding of the molecular mechanisms underlying *LncHrt-*mediated cardiac protection following MI, we performed genome-wide RNA sequencing (RNA-seq) on ventricular tissue from AAV9-mediated *LncHrt* overexpression (AAV-LncHrt) and control (AAV-CTRL) hearts 7 days post-MI (Fig. 4a). Boxplot, hierarchical clustering of the RNA-sequencing data, and PCA analysis confirmed consistency between the biological repeats for each group (*n *= 3 for each experimental group. Fig. S5, a–c). Hierarchical clustering analyses indicated that overexpression of *LncHrt* markedly altered the cardiac transcriptome following MI (Fig. 4b).

We identified 1763 differentially expressed genes (DEGs, adjust *P* value < 0.05) between the *LncHrt* overexpression and control group subjected to MI, in which 1083 genes were upregulated and 680 genes were downregulated (Fig. 4c). We then analyzed the functional annotation of these differentially expressed genes using Database for Annotation and Visualization and Integrated Discovery (DAVID) tools. Kyoto Encyclopedia of Genes and Genomes (KEGG) pathway analyses revealed upregulated genes are largely related to metabolic-related pathways, namely “metabolic pathways”, “carbon metabolism”, “oxidative phosphorylation”, “the citric acid (TCA) cycle”, “glycolysis”, and “fatty acid metabolism”. The “cardiac muscle contraction” related pathway was also upregulated. In contrast, downregulated genes are related to cardiac pathological pathways, such as “ECM-receptor interaction” and “focal adhesion” (Fig. 4d). Similarly, Gene Ontology (GO) biological process analyses confirmed that “metabolic processes” and “cardiac muscle contraction” are enriched among upregulated genes while “immune system process”, “inflammatory response”, and “collagen fibril organization” are enriched in downregulated genes (Fig. 4e).

These findings indicate that *LncHrt* overexpression-improved heart function post-Ml likely, by mediating cardiac metabolic pathways. To test this hypothesis, we performed comparative analyses of transcriptome signatures from both control and MI hearts with or without *LncHrt* overexpression (Fig. 4f). Gene Set Enrichment Analysis (GSEA) demonstrated that MI-induced down-regulation of extensive metabolic processes such as “oxidative phosphorylation”, “fatty acid metabolism”, and “glycolysis” were completely restored upon *LncHrt* overexpression compared to control post-MI (Fig. 4g). Cardiac pathological pathways like “ECM receptor interaction” and “TGF beta signaling pathway” were repressed by *LncHrt* in MI hearts and “cardiac muscle contraction” was increased (Fig. 4f and Fig. S5d). These analyses support the notion that *LncHrt* plays an important role in cardiac metabolism and muscle contraction following MI.

To confirm these results experimentally, we collected heart samples from an independent set of animals and performed qRT-PCR analysis for differentially expressed signature genes. Our results validated the changes described above in multiple pathways including “carbon metabolism”, “TCA cycle”, “fatty acid metabolism”, “glycolysis”, “fatty acid degradation”, “pyruvate metabolism”, and “cardiac muscle contraction” (Fig. 4h). In contrast, knock-down of *LncHrt* resulted in an opposite effect on metabolic gene expression (Fig. S5e). Together, these data indicate *LncHrt* positively modulates metabolic-related gene expression, enhanced cardiac muscle contraction, and suppressed cardiac fibrosis, which may improve cardiac function post-MI.

### *LncHrt* improved cardiac metabolic homeostasis post-MI

**Fig. 5:**
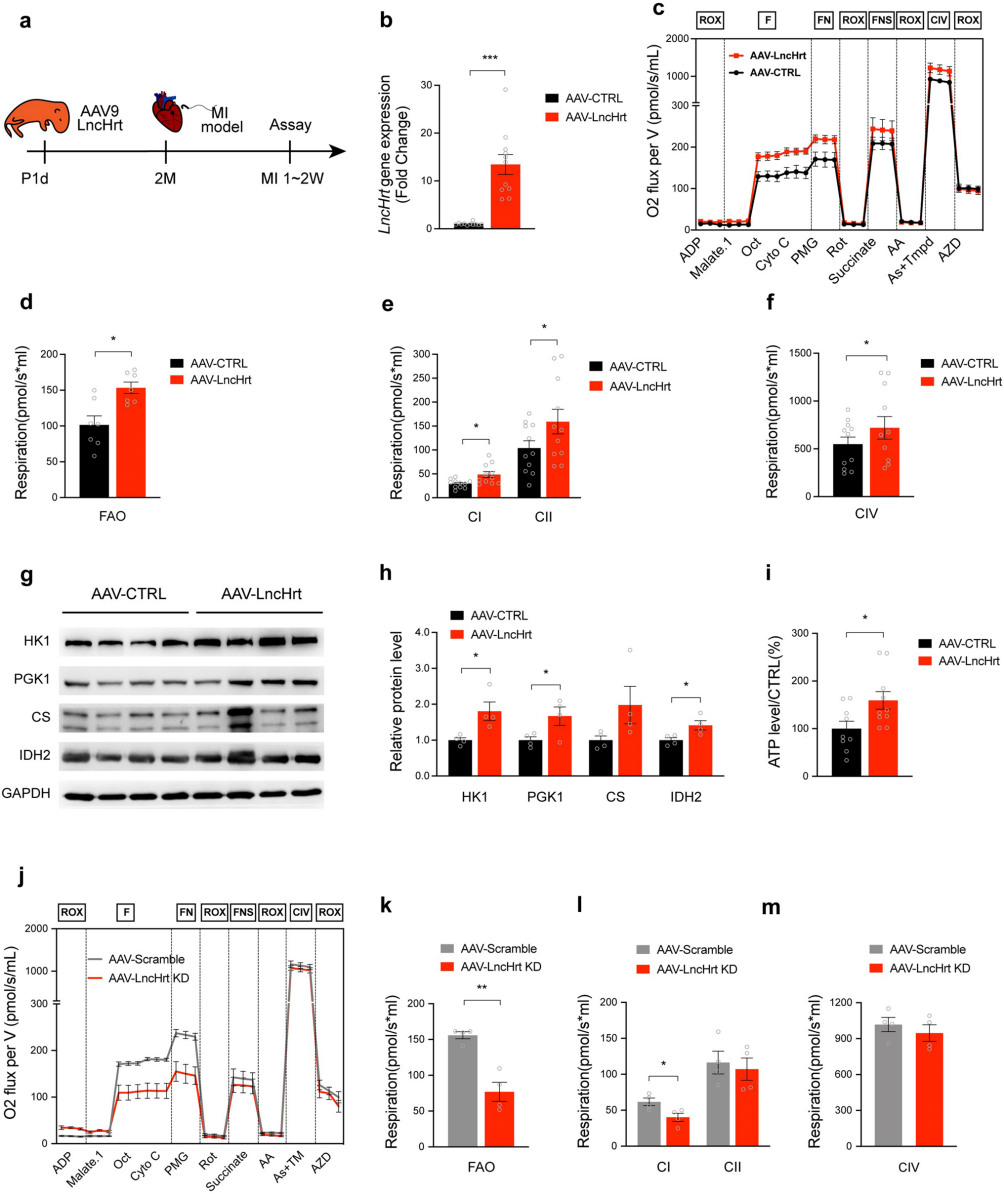
*LncHrt* improved cardiac metabolic homeostasis post myocardial infarction. a Schematic plot showing the experimental design with AAV9 delivered *LncHrt* overexpression in mice heart with myocardial infarction injury, mitochondrial respiration capacity and glucose utilization was performed from 1 to 2 weeks post-MI (MI-1 W and MI-2 W). **b** qRT-PCR of LncHrt gene expression between MI-1 W and MI-2 W. *Rn18S* as a reference gene. Data are mean ± s.e.m. *P < 0.05 and **P < 0.01 (versus AAV-CTRL); by Mann–Whitney test, *n *= 11 mice per group. **c** Representative respiratory experiment of mitochondrial oxidative phosphorylation (OXPHOS) capacity of complex with fatty acid oxidation pathway(F-pathway) in OXPHOS state by using application of substrate uncoupler inhibitor titration (SUIT) protocols to interrogate sequentially different substrate and coupling states using saponin permeabilized myofibers. **d** Fatty acid oxidation (FAO) as measured by oxygen consumption with octanoylcarnitine corrected to corresponding residual oxygen consumption (ROX). Data are mean ± s.e.m. *P < 0.05 and **P < 0.01 (versus AAV-CTRL); by Student’s* t *test, *n *= 7 mice per group. **e** Combined analysis of mitochondrial oxidative phosphorylation (OXPHOS) capacity of complex I (CI) and complex II (CII) in electron transfer (ET) state with or without fatty acid oxidation pathway(F-pathway) in OXPHOS state. The oxygen consumption rate (OCR) of complex I showed oxygen consumption with Pyruvate (P), Malate (M) and Glutamate (G) (PMG) titrations minus octanoylcarnitine (OCT) titrations or corrected to corresponding residual oxygen consumption (ROX), while complex II respiration rate corrected to corresponding ROX. Data are mean ± s.e.m. *P < 0.05 and **P < 0.01 (versus AAV-CTRL); by Student’s* t *test, *n *= 11 mice per group. **f** Combined analysis of mitochondrial oxidative phosphorylation (OXPHOS) capacity of complex IV (CIV) in electron transfer (ET) state with or without fatty acid oxidation pathway(F-pathway) in OXPHOS state. As illustrated by oxygen consumption rate (OCR) corrected to corresponding residual oxygen consumption (ROX). Data are mean ± s.e.m. *P < 0.05 and **P < 0.01 (versus AAV-CTRL); by Student’s* t *test, *n *= 11 mice per group. **g** Western blot of key enzymes of glycolysis and TCA cycle in infarcted myocardium. **h** Quantification of Western blot band density using image J. Data are mean ± s.e.m. *P < 0.05 and **P < 0.01 (versus AAV-CTRL); by Student’s* t *test, *n *= 4 mice per group. **i** ATP content in infarct heart tissues normalize to protein levels. Data are mean ± s.e.m. *P < 0.05 and **P < 0.01 (versus AAV-CTRL); by Student’s* t *test, *n *= 9–10 mice per group. **j** Representative respiratory experiment of mitochondrial oxidative phosphorylation (OXPHOS) capacity of complex with fatty acid oxidation pathway(F-pathway) in OXPHOS state by using application of substrate uncoupler inhibitor titration (SUIT) protocols to interrogate sequentially different substrate and coupling states using saponin permeabilized myofibers (AAV-LncHrt KD versus AAV-Scramble). **k** Fatty acid oxidation (FAO), as measured by oxygen consumption with octanoylcarnitine corrected to corresponding residual oxygen consumption (ROX). Data are mean ± s.e.m. *P < 0.05 and **P < 0.01 (versus AAV-Scramble); by Student’s* t *test, *n *= 4 mice per group. **l** Mitochondrial respiration of complex I (CI) and complex II (CII). The oxygen consumption rate (OCR) of complex I showed oxygen consumption with Pyruvate (P), Malate (M) and Glutamate (G) (PMG) titrations minus octanoylcarnitine titrations, while complex II respiration rate corrected to corresponding residual oxygen consumption (ROX). Data are mean ± s.e.m. *P < 0.05 and **P < 0.01 (versus AAV-Scramble); by Student’s* t *test, *n *= 4 mice per group. **m** Mitochondrial oxidative phosphorylation (OXPHOS) capacity of complex IV (CIV). As illustrated by oxygen consumption rate (OCR) corrected to corresponding residual oxygen consumption (ROX). Data are mean ± s.e.m. *P < 0.05 and **P < 0.01 (versus AAV-Scramble); by Student’s* t *test, *n *= 4 mice per group

The metabolic processes modulated by *LncHrt* post-MI identified by RNA-seq analysis prompted us to investigate how *LncHrt* mitigates cardiac metabolic stress and improve metabolic phenotype in response to cardiac remodeling and stress (Fig. 5a). Cardiac *LncHrt* overexpression was confirmed by RT-PCR in mouse hearts after AAV-LncHrt injection (Fig. 5b). We assessed mitochondrial respiration by measuring oxygen consumption rate (OCR) in permeabilized mouse myofibers in the remote ischemic zone using high-resolution respirometry following substrate-uncoupler-inhibitor-titration (SUIT) protocols [34, 41]. ADP-stimulated oxygen flux was determined under coupled conditions to estimate mitochondrial function, while oxygen flux was enhanced in *LncHrt*-injected hearts (Fig. 5c and Fig. S6a). Respiratory stimulation of the fatty acid oxidation (FAO) pathway in the presence of malate (M) and octanoylcarnitine (Oct) was significantly elevated by *LncHrt* (Fig. 5d). Combined analyses of the oxidative phosphorylation (OXPHOS) capacity of mitochondria in an electron transfer (ET) state, with or without fatty acid oxidation (F-pathway) were performed (Fig. 5c and Fig. S6a). *LncHrt* overexpression significantly promoted complex I OXPHOS capacity in the presence of pyruvate (P), glutamate (G), and malate (M) to 165%, and the complex II oxygen consumption rate (OCR) increased to 154%, when compared to the CTRL group, respectively (Fig. 5e). By adding ascorbate (As) and TMPD, the respiration of complex IV was increased to 131% in *LncHr*t-overexpressing hearts, when compared with controls (Fig. 5f). We also assessed mitochondrial content by examining the protein level of the mitochondrial proteins, VDAC and COXIV. We found that *LncHrt* overexpression did not alter the mitochondrial content, indicating the beneficial effect of *LncHrt* on mitochondrial respiration were not from a higher content of mitochondria in the preparations used for respiration measurement (Fig. S6, b, c). Together, these studies indicate cardiac overexpression of *LncHrt* improves oxidative phosphorylation metabolic capacity in the heart after MI.

Given that reductions in the supply of oxygen following MI elicits a metabolic shift to glucose utilization [29], we examined the protein levels of hexokinase-1 (HK1) [16] and phosphoglycerate kinase 1 (PGK1) [38], which are both key glycolytic enzymes, as well as citrate synthase (CS) [8, 23], isocitrate dehydrogenase (IDH2) [31], and energy production enzymes in the infarct of hearts under hypoxic conditions. We found a marked increase in the expression of these proteins in *LncHrt* overexpressing hearts (Fig. 5g, h). These results indicate that *LncHrt* overexpression protected mitochondrial oxidative metabolism after MI.

Next, we determined if *LncHrt* increased cardiac ATP content after MI. *LncHrt* overexpression markedly enhanced ATP levels by 160% compared to CTRL hearts after MI (Fig. 5i). Conversely, we asked whether knock-down of *LncHrt* also impacts metabolic homeostasis in the heart. We measured key metabolic pathways involved in cardiac contraction using permeabilized mouse myofibers. As expected, we observed impaired mitochondrial respiration (Fig. 5j), fatty acid oxidation (Fig. 5k), and complex I oxidative phosphorylation capacity in the *LncHrt* knockdown group compared to the control group with mild inhibition of complex II and complex IV oxidative phosphorylation capacity (Fig. 5l, m). Together, our results show *LncHrt* preserved cardiac metabolic homeostasis and improved cardiac function after MI.

### *LncHrt* activates the LKB1-AMPK signaling pathway via SIRT2

To elucidate the molecular mechanism by which *LncHrt* modulates myocardial metabolic homeostasis in response to MI, we first asked whether *LncHrt* may regulate the expression of its neighboring gene, *Klhl33*. We overexpressed *LncHrt* to assess the transcriptional impact on *Klhl33 *in vivo and in vitro. qRT-PCR analysis showed that *LncHrt* overexpression had no effect on *Klhl33* gene expression in vivo before (Fig.S7a) or after MI (Fig.S7b) and overexpression of *LncHrt* did not affect *Klhl33* levels in vitro (Fig.S7c).

**Fig. 6:**
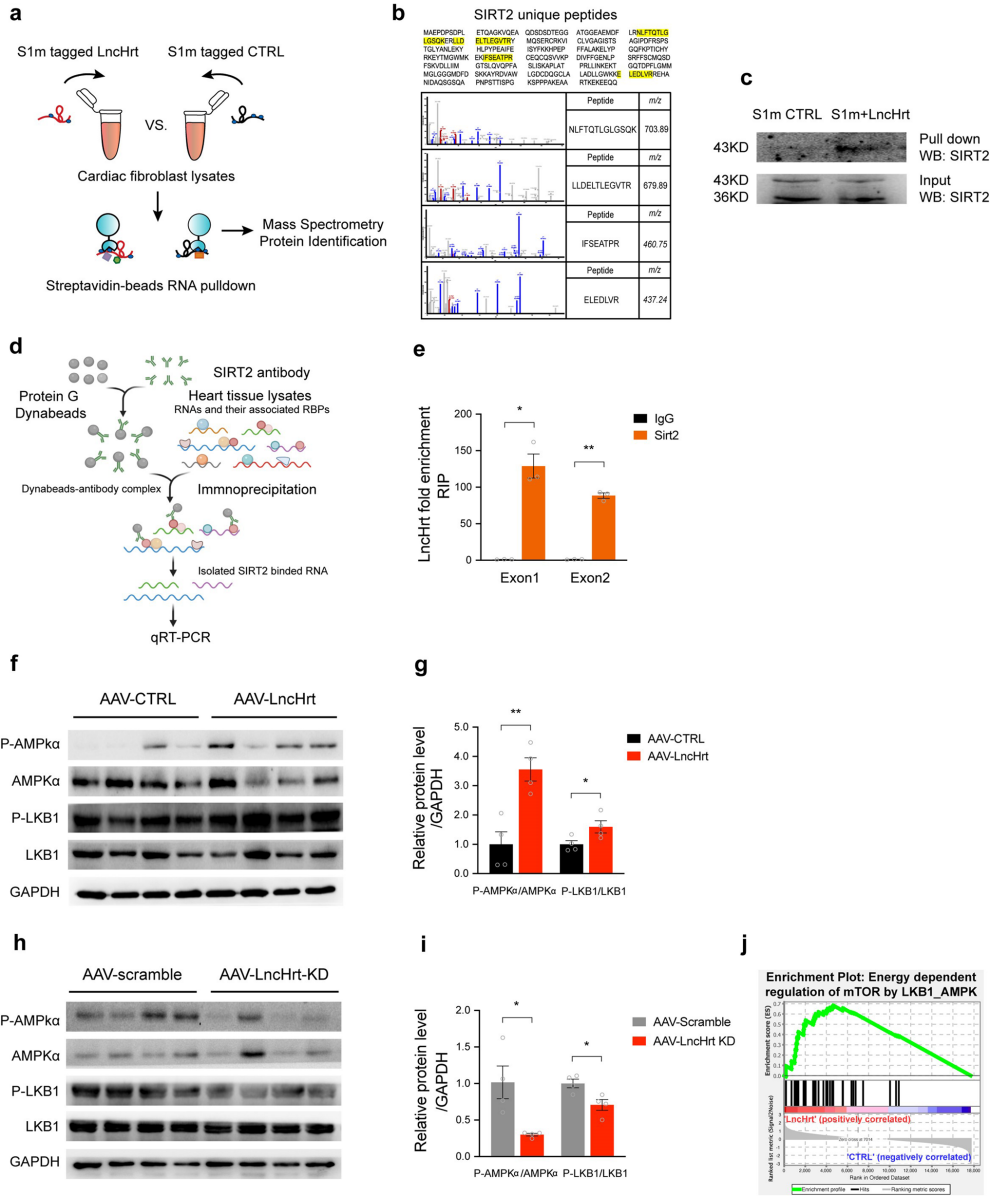
*LncHrt* activates the LKB1-AMPK signaling pathway by interacting with SIRT2. **a** Workflow of S1m tagged *LncHrt* pull down assay in cardiac cells. **b** Amino acid of the protein SIRT2. The detected peptide by mass spectrometry is highlighted in yellow. **c** Western blot of SIRT2 protein in S1m tagged LncHrt vs. S1m CTRL pull down in cardiomyocytes. **d** Workflow of endogenous *LncHrt* immunoprecipitation (RIP) by SIRT2 antibody. **e** SIRT2 was immunoprecipitated from heart tissue and co-precipitated *LncHrt* were detected by qRT–PCR using 2 primers for *LncHrt*. IP enrichment is determined as the amount of RNA associated to SIRT2 IP relative to IgG control. Data are mean ± s.e.m. *P < 0.05 and **P < 0.01; by Student’s* t *test, *n *= 3 independent assays. **f** Western blot of LKB1-AMPK pathway in AAV-LncHrt injected heart tissues post myocardial infarction. **g** Quantification of protein expression of LKB1-AMPK pathway. Data are mean ± s.e.m. *P < 0.05 and **P < 0.01 (versus AAV-CTRL); by Student’s* t *test, *n *= 4 mice per group. **h** Western blot of LKB1-AMPK pathway in AAV-LncHrt shRNA injected heart tissues. **i** Quantification of protein expression of LKB1-AMPK pathway in AAV-LncHrt shRNA injected heart tissues. Data are mean ± s.e.m. *P < 0.05 and **P < 0.01 (versus AAV-CTRL); by Student’s* t *test, *n *= 4 mice per group. **j** The enrichment plot of energy dependent regulation of mTOR by LKB1-AMPK pathway by GSEA analysis

We identified *LncHrt*-interacting proteins by fusing a modified streptavidin (SA)-binding S1 RNA tag, termed S1m [37], to full-length *LncHrt* and expressing this construct in cardiac fibroblasts. The S1m construct lacking *LncHrt* served as a negative control. Then, streptavidin-conjugated magnetic beads were used in an RNA pull-down assay, and putative LncHrt-interacting proteins were identified by mass spectrometry (Fig. 6a). From this screening, we found many putative *LncHrt*-interacting proteins. At the top of this list is NAD-dependent deacetylase sirtuin 2, encoded by the *Sirt2* gene (Fig. 6b and Supplementary Table 6). We investigated SIRT2 because it is a crucial metabolic modulator that acts as a cardioprotective deacetylase in pathological hypertrophy [53, 58, 72]. We validated that *LncHrt* interacted with SIRT2 in cardiomyocytes using a S1m-fused *LncHrt* pull-down assay (Fig. 6c). To confirm the interaction between *LncHrt* and SIRT2 endogenously, we performed an RNA immunoprecipitation (RIP) assay. Anti-SIRT2 antibody was incubated with heart tissue lysates and co-precipitated *LncHrt* was analyzed by qRT-PCR, using two primer sets to detect two independent exons of the *LncHrt* gene. We observed enrichment of *LncHrt* in SIRT2 immunoprecipitate compared to IgG control (Fig. 6d, e), indicating an association between *LncHrt* and SIRT2 in the heart.

We then investigated the functional relevance of this interaction. A previous study showed that SIRT2 deacetylates downstream kinase LKB1 promoting the phosphorylation of LKB1 and subsequently activating LKB1-AMPK signaling. This pathway is cardioprotective by preserving the activity of AMPK in hypertrophic hearts [58]. As a result, we assessed whether *LncHrt* regulated LKB1-AMPK activation, and we found overexpression of *LncHrt* promoted LKB1 phosphorylation, which results downstream kinase AMPK phosphorylation at Thr172. The activation of the SIRT2 LKB1-AMPK kinase cascade by *LncHrt* (Fig. 6f, g) contrasts with *LncHrt* knock-down decreasing the phosphorylation of LKB1 and AMPK, which inhibits LKB1-AMPK signaling (Fig. 6h, i). These data are supported by our Gene Set Enrichment Analysis from the RNA-seq data of *LncHrt* overexpressing hearts, where energy-dependent regulation of mTOR by the LKB1-AMPK pathway is enriched (Fig. 6j). Together, these studies show that *LncHrt* interacts with SIRT2 to regulate its function by modulating LKB1-AMPK signaling in the infarct heart.

### *LncHrt* preserves SIRT2 activity by blocking CDK5 inhibition

**Fig. 7:**
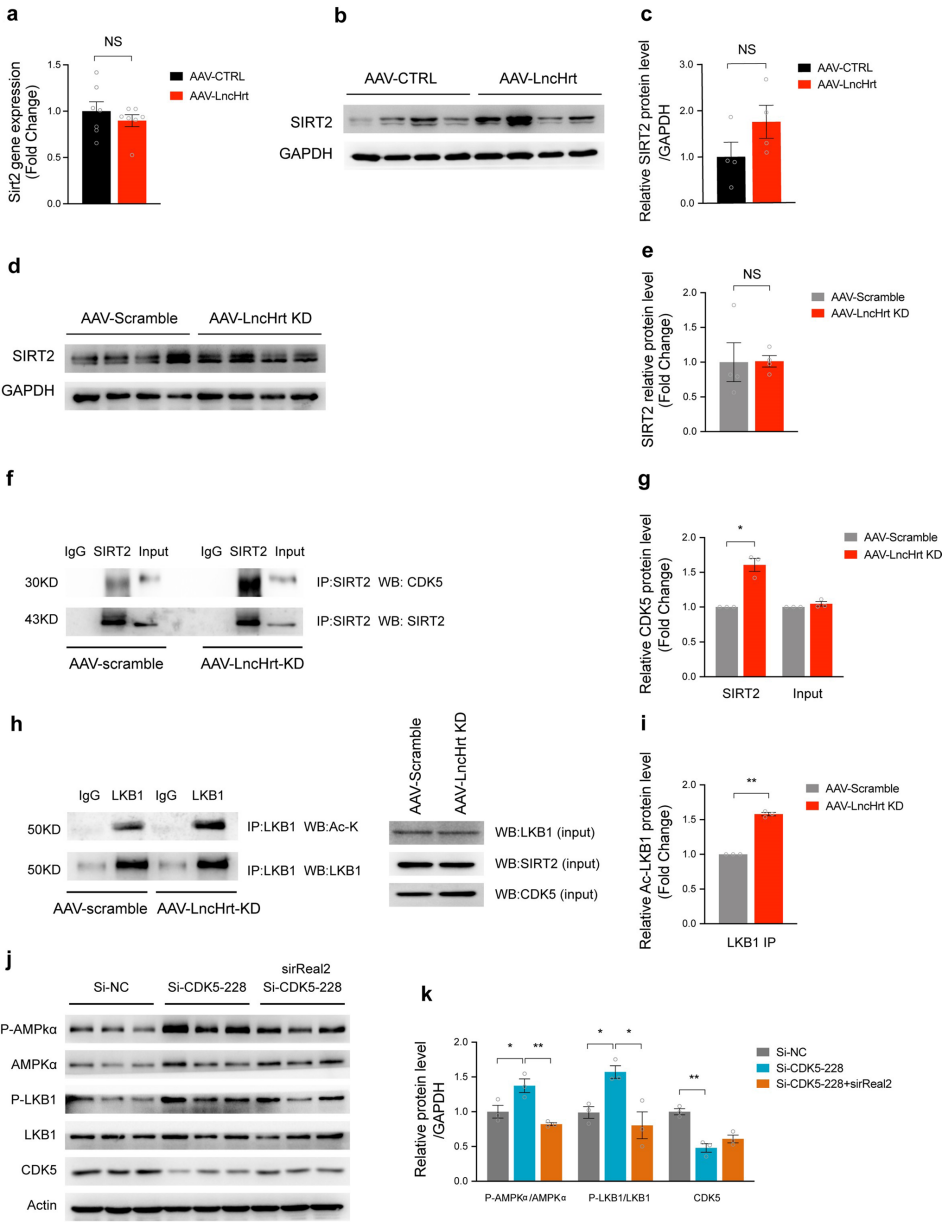
*LncHrt* preserved SIRT2 activity by blocking CDK5 inhibition on SIRT2. **a** qRT-PCR of *Sirt2* gene expression in AAV-LncHrt injected heart tissues post-MI. *Rn18S* as a reference gene. Data are mean ± s.e.m. *P < 0.05 and **P < 0.01 (versus AAV-CTRL); by Student’s* t *test, *n *= 4 mice per group. **b** Western blot of SIRT2 protein in AAV-LncHrt injected heart tissues (versus AAV-CTRL) post-MI. **c** Quantification of protein expression of SIRT2. Data are mean ± s.e.m. *P < 0.05 and **P < 0.01 (versus AAV-CTRL); by Student’s* t *test, *n *= 4 mice per group. **d** Western blot of SIRT2 protein in AAV-LncHrt KD (versus AAV-Scramble) heart tissues. **e** Quantification of protein expression of SIRT2. Data are mean ± s.e.m. *P < 0.05 and **P < 0.01 (versus AAV-Scramble); by Student’s* t *test, *n *= 4 mice per group. **f** Western blot of co-immunoprecipitated CDK5 and SIRT2 using anti-SIRT2 antibodies with negative control IgG antibody in scramble and LncHrt knock-down heart tissue lysates. **g** Quantification of protein enrichment of co-immunoprecipitated CDK5 in scramble and LncHrt knock-down heart tissue lysates. Data are mean ± s.e.m. *P < 0.05 and **P < 0.01; by Student’s* t *test, *n *= 3 independent assays. **h** Western blot of acetylated lysine and immunoprecipitated LKB1 using anti-LKB1 antibodies with negative control IgG antibody in scramble and *LncHrt* knock-down heart tissue lysates. **i** Quantification of acetylated LKB1 enrichment in scramble and *LncHrt* knock-down heart tissue lysates. Data are mean ± s.e.m. *P < 0.05 and **P < 0.01; by Student’s* t *test, *n *= 3 independent assays. **j** Western blot of LKB1-AMPK pathway in hypoxic neonatal cardiomyocytes (1% O2 treatment for 24 h) treated with CDK5 siRNA and SirReal2. **k** Quantification of protein expression of LKB1-AMPK pathway in hypoxic cardiomyocytes with various treatments. Data are mean ± s.e.m. *P < 0.05 and **P < 0.01; by One-way ANOVA analysis, *n *= 3

As *LncHrt* preserved SIRT2 activity and promoted downstream kinase activity, we asked how *LncHrt* impacts the SIRT2 upstream signals to positively regulate its activity. We first asked if *LncHrt* influences *Sirt2* mRNA or SIRT2 protein levels. We established that *LncHrt* did not affect *Sirt2* transcript levels or protein expression since Sirt2 was not changed in the *LncHrt* gain-of-function (Fig. 7a–c) or loss-of-function models (Fig. 7d, e).

We then investigated the possibility that *LncHrt* promotes SIRT2 activity through post-translational regulation. A previous study revealed a post-translational mechanism by which p35-Cdk5 represses the catalytic activity of SIRT2 by S331 phosphorylation in post-mitotic cells [48]. Given AAV9-delivered *LncHrt* specifically overexpressed in cardiomyocytes, we hypothesized that CDK5 post-translationally modifies SIRT2, which suppresses LKB1-AMPK activity. We first confirmed the interaction of CDK5 with SIRT2 in the heart by co-IP (Fig. S8a). Intriguingly, we found that knock-down of *LncHrt* promoted the interaction between CDK5 and SIRT2 (Fig. 7f, g), and, subsequently, elevated the lysine acetylation of LKB1, resulting in abrogation of LKB1 activity and its downstream signaling cascades (Fig. 7h, i). However, *LncHrt* appeared to have no effect on the protein level of CDK5 in either an overexpression or depletion setting (Fig. S8, b–e). This observation ruled out the idea that *LncHrt* directly modulated the expression of CDK5.

Finally, we asked whether depletion of CDK5 modulates SIRT2 downstream of LKB1-AMPK signaling in cardiomyocytes. We found that knock-down of CDK5 boosted LKB1-AMPK signaling, while SIRT2 inhibition with SirReal2 effectively blocked the LKB1-AMPK activation (Fig. 7j, k). Consequently, we confirmed CDK5-mediated LKB1-AMPK signaling by modulating SIRT2 activity. *LncHrt* exhibited cardiac metabolic protection, at least, in part, by interfering with the catalytic activity of SIRT2, leading to LKB1-AMPK signal activation.

### Therapeutic potential of *LncHrt* in infarcted hearts

Having demonstrated *LncHrt* protects the heart from MI injury, we next asked whether *LncHrt* could serve as potential therapeutic target to treat infarct hearts. We performed intra-myocardial injection of AAV9-LncHrt or AAV9-CTRL (2 ~ 5 × 10^11^ viral genome particles per mouse heart), respectively, post-MI. We carefully examined *the LncHrt* gene expression at 1 day, 3 days, 9 days, and 6 weeks post-MI. Similar to our previous study of using AAV-delivery [12], the expression level of *LncHrt* was not altered until 3 days after MI with a mild increase and appeared full expressed by 9 days post-MI and maintained for 6 weeks post-MI (*i.e.*, when all cardiac parameters were collected) (Fig. S9a).

**Fig. 8:**
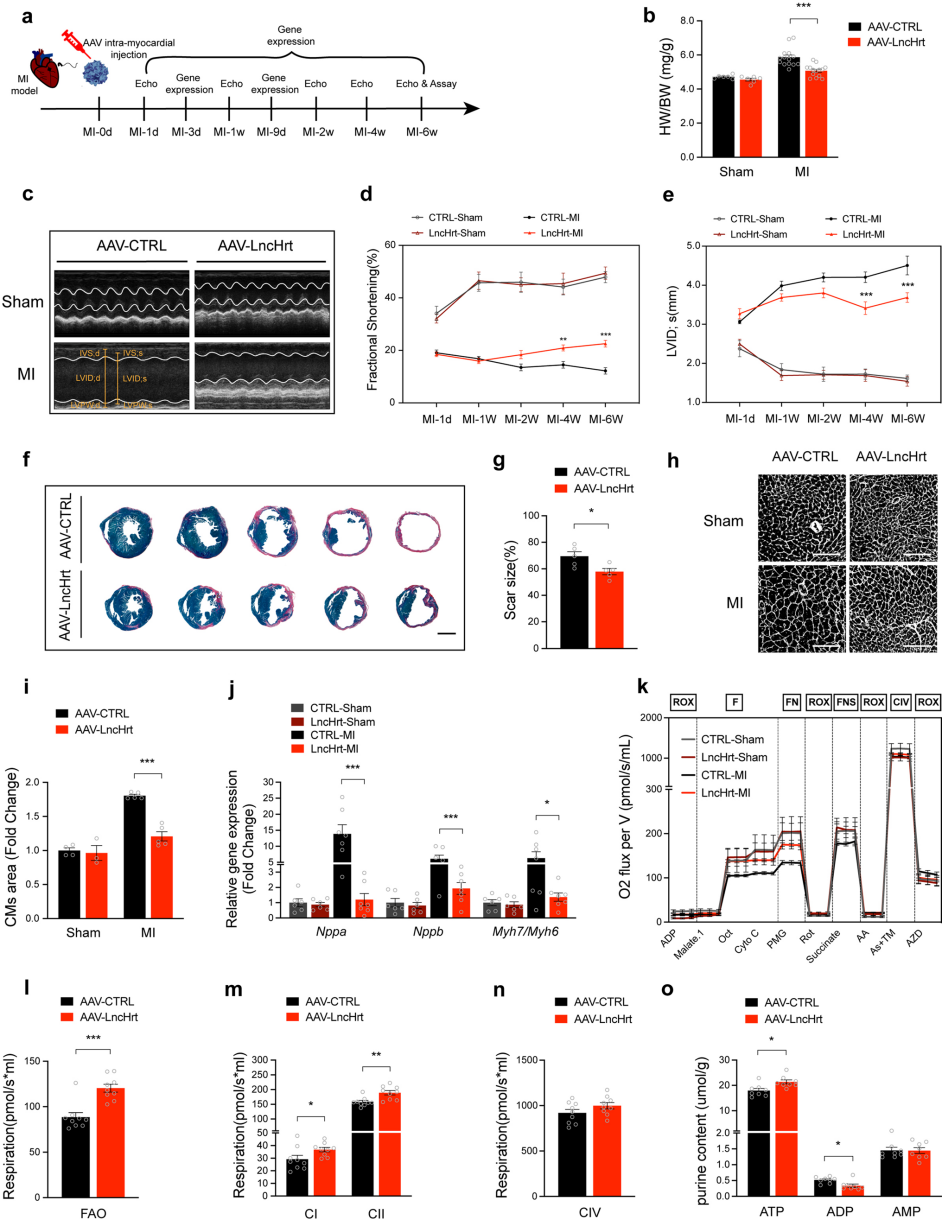
Therapeutic potential of *LncHrt* in infarcted hearts. **a** Schematic diagram showing the experimental design with AAV9-delivered *LncHrt* therapeutic potential in infarcted hearts. *LncHrt* gene expression was examined 1 day, 3 day, 9 day and 6 weeks after MI. Echocardiography was performed at post-MI 1 day, 1 week, 2 weeks, 4 weeks and 6 weeks. **b** Ratio of heart weight (HW) to body weight (BW) in AAV-CTRL and AAV-LncHrt heart post-MI (2 months). Data are mean ± s.e.m. *P < 0.05 and **P < 0.01 (versus AAV-CTRL); by two-way ANOVA analysis, *n *= 6 mice in sham group and *n *= 14 mice in MI group. **c** M-mode echocardiography of mice heart 6 weeks post-MI. **d** Analyses of cardiac function of FS% after AAV-LncHrt intra-myocardial injection at 1 day, 1 week, 2 weeks, 4 weeks and 6 weeks post-MI compared to their CTRL group. FS, left ventricular fractional shortening. Data are mean ± s.e.m. *P < 0.05 and **P < 0.01 (versus AAV-CTRL); by two-way ANOVA analysis, *n *= 6 mice in sham group and *n *= 17 mice in MI group. **e** Analyses of cardiac function of LVID after AAV-LncHrt intra-myocardial injection at 1 day, 1 week, 2 weeks, 4 weeks and 6 weeks post-MI compared to their CTRL group. LVIDs, LV internal dimension at end-systole. Data are mean ± s.e.m. *P < 0.05 and **P < 0.01 (versus AAV-CTRL); by two-way ANOVA analysis, *n *= 6 mice in sham group and *n *= 17 mice in MI group. **f** Representative of images of Sirius red/fast green collagen staining of series of transverse sections after injection of AAV-LncHrt compared to AAV-CTRL group after MI injury. Sirius red/fast green collagen staining marks myocardium (green) and scar (red). Scale bars = 2 mm. **g** Quantification of scar size of AAV-LncHrt compared to AAV-CTRL group after MI injury. Data are mean ± s.e.m. *P < 0.05 and **P < 0.01 (versus AAV-CTRL); by Student’s* t *test, *n *= 5 mice per group. **h** Representative images of cardiomyocytes stained with WGA. Scale bars = 100 μm. **i** Quantification of cardiomyocytes area of AAV-LncHrt compared to AAV-CTRL group after MI injury. Data are mean ± s.e.m. *P < 0.05, **P < 0.01 and ***P < 0.001 (versus AAV-CTRL); by two-way ANOVA analysis, *n *= 3 or 5 mouse hearts per group, measured ~ 200 cardiomyocytes per heart and calculated median value for each heart. **j** Hypertrophic marker gene expression of heart of AAV-LncHrt versus AAV-CTRL group post sham and MI. *Rn18S* as a reference gene. Data are mean ± s.e.m. *P < 0.05 and **P < 0.01 (versus AAV-CTRL); by two-way ANOVA analysis, *n *= 6 mice in sham group and *n *= 7 mice in MI group. **k** Representative respiratory experiment of mitochondrial oxidative phosphorylation (OXPHOS) capacity of complex with fatty acid oxidation pathway(F-pathway) in OXPHOS state by using application of substrate uncoupler inhibitor titration (SUIT) protocols to interrogate sequentially different substrate and coupling states using saponin permeabilized myofibers (AAV-LncHrt versus AAV- CTRL post sham and MI). **l** Fatty acid oxidation (FAO) after MI injury, as measured by oxygen consumption with octanoylcarnitine corrected to corresponding ROX. Data are mean ± s.e.m. *P < 0.05 and **P < 0.01 (versus AAV-CTRL post-MI); by Student’s* t *test, *n *= 9 mice per group. **m** Mitochondrial respiration of complex I (CI) and complex II (CII) after MI injury. The oxygen consumption rate (OCR) of complex I showed oxygen consumption with Pyruvate (P), Malate (M) and Glutamate (G) (PMG) titrations minus octanoylcarnitine titrations, while complex II respiration rate corrected to corresponding ROX. Data are mean ± s.e.m. *P < 0.05 and **P < 0.01 (versus AAV-CTRL post-MI); by Student’s* t *test, *n *= 9 mice per group. **n** Mitochondrial OXPHOS capacity of complex IV (CIV) after MI injury. As illustrated by OCR corrected to corresponding ROX. Data are mean ± s.e.m. *P < 0.05 and **P < 0.01 (versus AAV-CTRL post-MI); by Student’s* t *test, *n *= 9 mice per group. **o** Purine content in infarct heart tissues normalize to protein levels. Data are mean ± s.e.m. *P < 0.05 and **P < 0.01 (versus AAV-CTRL); by Student’s* t *test, *n *= 8 mice per group

We observed that *LncHrt* improved cardiac morphology and significantly reduced the HW/BW ratio in response to MI compared to the control group (Fig. 8b). Similar to the expression pattern, we noticed no difference in cardiac function between AAV-*LncHrt* and AAV-CTRL injected hearts at 24 h post-MI; however, cardiac function was improved 2 weeks after *LncHrt* injection, as determined by echocardiography with increased fractional shortening (FS%) from MI-2 W till to MI-6 W (Fig. 8c, d and Supplementary Table 7, 8), reduced cardiac chamber internal diameter (LVID,s (mm), Fig. 8e and Supplementary Table 7, 8).

Histological analyses revealed that *LncHrt* substantially reduced infarct fibrosis following MI when compared to controls (Fig. 8f, g) without induce cardiac fibrosis in sham hearts (Fig. S9. b, c). In addition, cardiomyocyte size was markedly decreased in AAV-LncHrt treated hearts post-MI, while no difference was observed in sham hearts (Fig. 8h, i). In addition, the expression of cardiac hypertrophic marker genes, *Nppa, Nppb,* and *Myh7/Myh6* was significantly reduced in AAV-LncHrt treated hearts post-MI, while no difference was observed in sham hearts (Fig. 8j). These results indicate that intra-myocardial injection of AAV9-LncHrt prevented cardiac maladaptive remodeling post-MI.

We further assessed the therapeutic effect of *LncHrt* on cardiac metabolic homeostasis by measuring the oxidative phosphorylation (OXPHOS) capacity of permeabilized mouse myofibers (Fig. 8k). Respiratory stimulation of the fatty acid oxidation (FAO) pathway in the presence of malate (M) and octanoylcarnitine (Oct) was significantly elevated by *LncHrt* treatment post-MI (Fig. 8l), while no alteration was observed in the sham group (Fig. S9d). AAV-LncHrt also significantly promoted complex I OXPHOS capacity in the presence of pyruvate (P), glutamate (G) and malate (M), and the complex II substrate succinate oxygen consumption rate (OCR) (Fig. 8m), and tended to promote the respiration of complex IV (Fig. 8n) post-MI, while no difference was observed in sham groups (Fig. S9e and S9f). In addition, the results of purine content showed *LncHrt* significantly increased ATP content with decreased ADP content, indicating AAV-LncHrt treatment promotes ATP synthesis (Fig. 8o and Supplementary Table 9). However, no difference was found in the hearts of sham groups (Fig. S9g)*.* Together, these data demonstrate that *LncHrt* is a potential therapeutic target for treatment of myocardial infarction.

### Discussion

**Fig. 9:**
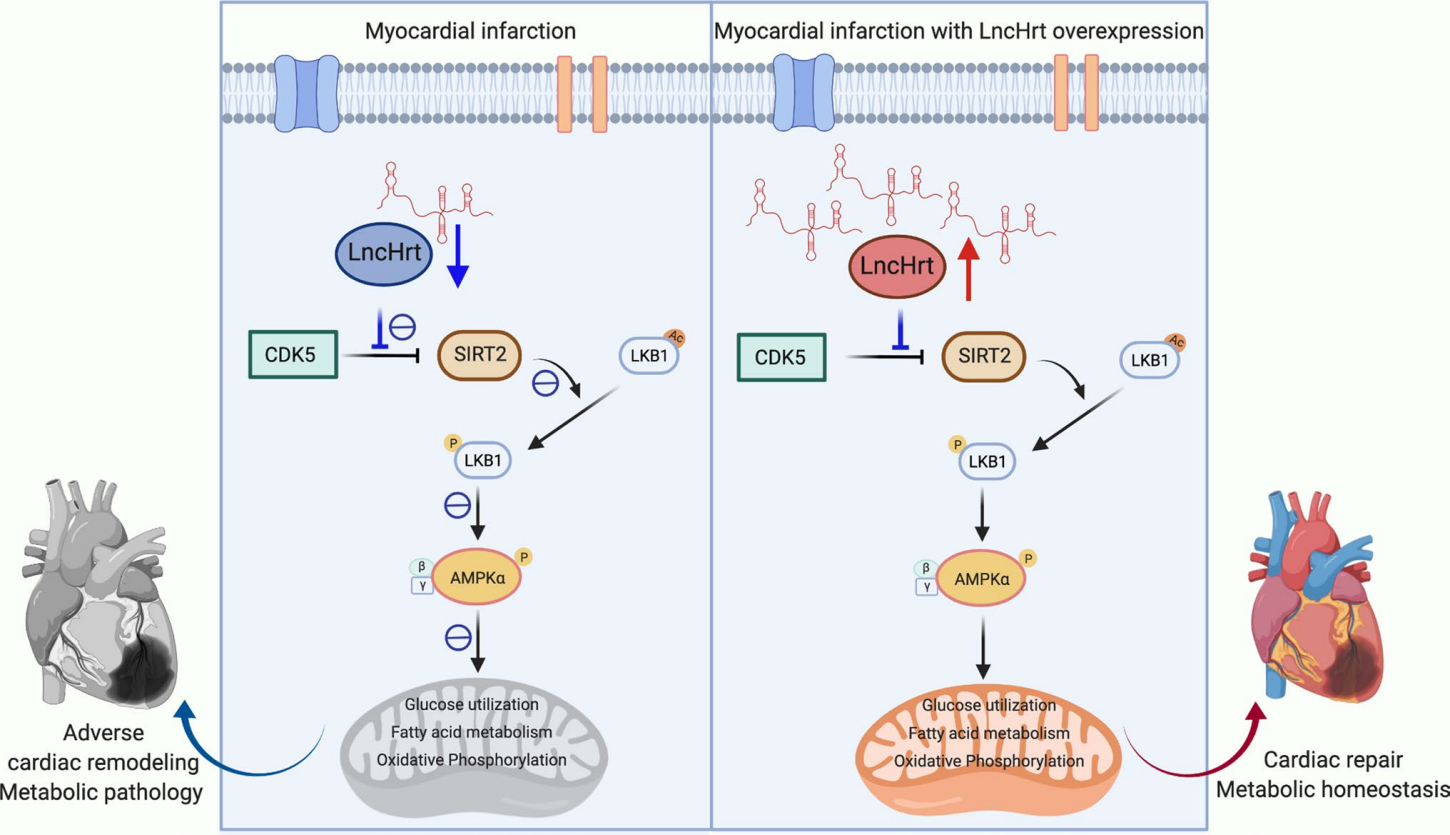
A working model summarizing the mechanism of *LncHrt* function in myocardial infarction. In the infarcted heart*, LncHrt* is repressed through an increased interaction between CDK5 and SIRT2 accompanied by inhibition of LKB1-AMPK signaling. Under compensated *LncHrt* gene expression, SIRT2 activity is preserved by interfering with CDK5 inhibition of catalytic activity, then SIRT2 deacetylates LKB1 and enhances phosphorylation of LKB1, which activates LKB1-AMPK signaling. This, in turn, preserves heart function and prevents adverse cardiac remodeling by modulating metabolic homeostasis

Here, we identified a novel cardiomyocyte-enriched lncRNA called *LncHrt*, which functions as an important regulator of cardiac metabolism. We found that *LncHrt* is the most down-regulated lncRNA in response to myocardial infarction (MI) and is essential for cardiac homeostasis, as depletion of *LncHrt* impairs cardiac morphology, metabolism, and function. Importantly, cardiac-specific overexpression of *LncHrt* protected the heart after MI through expression of genes involved in metabolic signaling and muscle contraction. These transcriptome alterations in *LncHrt-*overexpressing hearts compensated for MI-induced deregulation of metabolic signaling. Therefore, our study identified *LncHrt* as a novel cardiac lncRNA and has uncovered an unrecognized role for lncRNAs in maintaining cardiac metabolic homeostasis following pathological stress. In addition, we found the mechanism that couples lncRNAs with metabolism are mediated through the SIRT2-LKB1-AMPK axis, which provides evidence that lncRNAs can influence metabolic signaling cascades (Fig. 9).

Therapeutic strategies to attenuate adverse cardiac remodeling include increasing the supply of energy to the heart and switching the energy substrate preference to increase cardiac efficiency [14, 19]. In the infarcted heart, decreased mitochondrial respiration leads to decreased concentrations of ATP, resulting in compromised mitochondrial oxidation of fatty acids and carbohydrates, which impairs contractile function [11, 40, 44]. Promoting glucose utilization has been proposed as a means to increase ATP levels under hypoxic conditions [27]; however, decreased fatty acid oxidation is considered maladaptive [28, 54]. On the other hand, high fat diet-fed rats subjected to MI showed improved cardiac function through enhance fatty acid oxidation [2]. So, this metabolic phenotype affects recovery of the injured heart [2, 9]. With that in mind, we found that *LncHrt* not only accelerates glucose utilization in the infarcted myocardium, but also enhances mitochondrial respiration in intact cardiomyocytes from the non-infarcted area, which indicates *LncHrt* improves cardiac metabolism and protects the heart from ischemia.

During MI, AMPK is activated to maintain energy by increasing glycolysis and accelerating fatty acid oxidation [17]. Phosphorylation at Thr172 activates AMPK activity [69], which is mediated through LKB1 [67]. Deacetylation of LKB1 influences its intracellular localization, association with its partner protein STRAD, kinase activity, and its ability to activate AMPK [33]. Our study shows MI-induced downregulation of *LncHrt*. By overexpressing this lncRNA post-MI, AMPK activity can be increased to protect the ischemic heart. Therefore, *LncHrt* may function as an energy sensor.

The cyclin-dependent kinase CDK5 is important for neuronal development, survival, migration, differentiation, and metabolism [71]. CDK5 phosphorylates AMPKa2 at Thr485, which repressed its activity in the brain [39]. While we did not observe an interaction between CDK5 and AMPKa2 in the heart, CDK5 inhibition may be cardioprotective [24]. Suppressing CDK5 activity also alleviates the cardiac phenotype associated with Timothy Syndrome [55]. Although, we have shown that inhibition of CDK5 positively regulates LKB1 and AMPK activity, the pathophysiological role of this kinase has yet to be fully determined.

SIRT2 is the only sirtuin that is preferentially localized to the cytoplasm. SIRT2 plays an essential role in many aspects of metabolism [36, 58, 66, 72]. In particular, SIRT2 restrains cardiac hypertrophy by deacetylating LKB1 and activating LKB1-AMPK signaling [58]. CDK5 phosphorylates Ser331 in the C-terminal portion of the catalytic domain of SIRT2 to inhibit deacetylase activity of this protein, which affects neuronal adhesion, migration, and outgrowth [48]. In our study, we established that *LncHrt* regulated LKB1-AMPK activity by interfering with the CDK5/SIRT2 interaction and modulating cardiac metabolism. We speculate that manipulation of CDK5 activity has therapeutic potential for heart disease.

In this study, we demonstrated that *LncHrt* plays an important role in cardiac metabolic homeostasis post-MI through regulation of (1) metabolic gene expression; (2) mitochondrial respiration and (3) SIRT2-LKB1-AMPK signaling. However, we have not revealed these 3 *LncHrt*-modulated activities are connected and to what extent they contribute to cardioprotection after MI. Given that many studies have shown direct or indirect transcriptional regulation by AMPK, we speculate that upon *LncHrt*-stimulation of the SIRT2-LKB1-AMPK axis post-MI, activated AMPK controls metabolic gene expression through metabolites of intermediary metabolism or by modulating transcription factors and cofactors, such as CREB1 [6], PGC-1α, FOXO1 [4], and others [56]. Presumably, *LncHrt*-activated AMPK could transcriptionally modulate gene expression of metabolism and control energy homeostasis in cardiac disease. In addition, it is unclear whether *LncHrt*-mediated activation of the SIRT2-LKB1-AMPK axis is required to preserve mitochondrial respiration and/or prevent maladaptive remodeling after MI. As a result, a more complete view of *LncHrt*-regulated metabolic homeostasis (including signaling, mitochondrial respiration and gene expression) needs to be elucidated.

Most LncRNAs lack sequence conservation across species and the secondary structure of lncRNAs is more highly conserved than the RNA sequence [49, 59, 65]. However, we identified the gene locus of *LncHrt* in the human genome that is positionally conserved (*i.e.*, both reside on chromosome 14) and conserved with respect to its sequence (*i.e.*, in exon 1 and portions of exon 2 across multiple species). Combined analysis with MFE predicted the secondary structure of *LncHrt* with potential base-paring sites are also conserved. Furthermore, the expression of the human homolog of *LncHrt* is downregulated in patients with dilated cardiomyopathy. In view of that, we evaluated the therapeutic efficacy of AAV9-delivered *LncHrt* in mice as the effectiveness and safety of AAV-directed gene therapy has been proven in patients [42]. From this perspective, AAV9-delivery of *LncHrt* may represent a novel RNA-based therapy for ischemic heart disease.

## Supplementary Information

Below is the link to the electronic supplementary material.

Supplementary file1 (PDF 1557 KB)

Supplementary file2 (PDF 323 KB)
