# Supplementary material for: LncRNA LncHrt preserves cardiac metabolic homeostasis and heart function by modulating the LKB1-AMPK signaling pathway
Source: Basic Res Cardiol. 2021 Aug 11;116(1):48. doi: 10.1007/s00395-021-00887-3 (PMC8357683; doi:10.1007/s00395-021-00887-3)
Supplement: Supplementary file 1 — Supplementary file1 (PDF 1557 KB) [file 395_2021_887_MOESM1_ESM.pdf]

Supplementary Figure 1

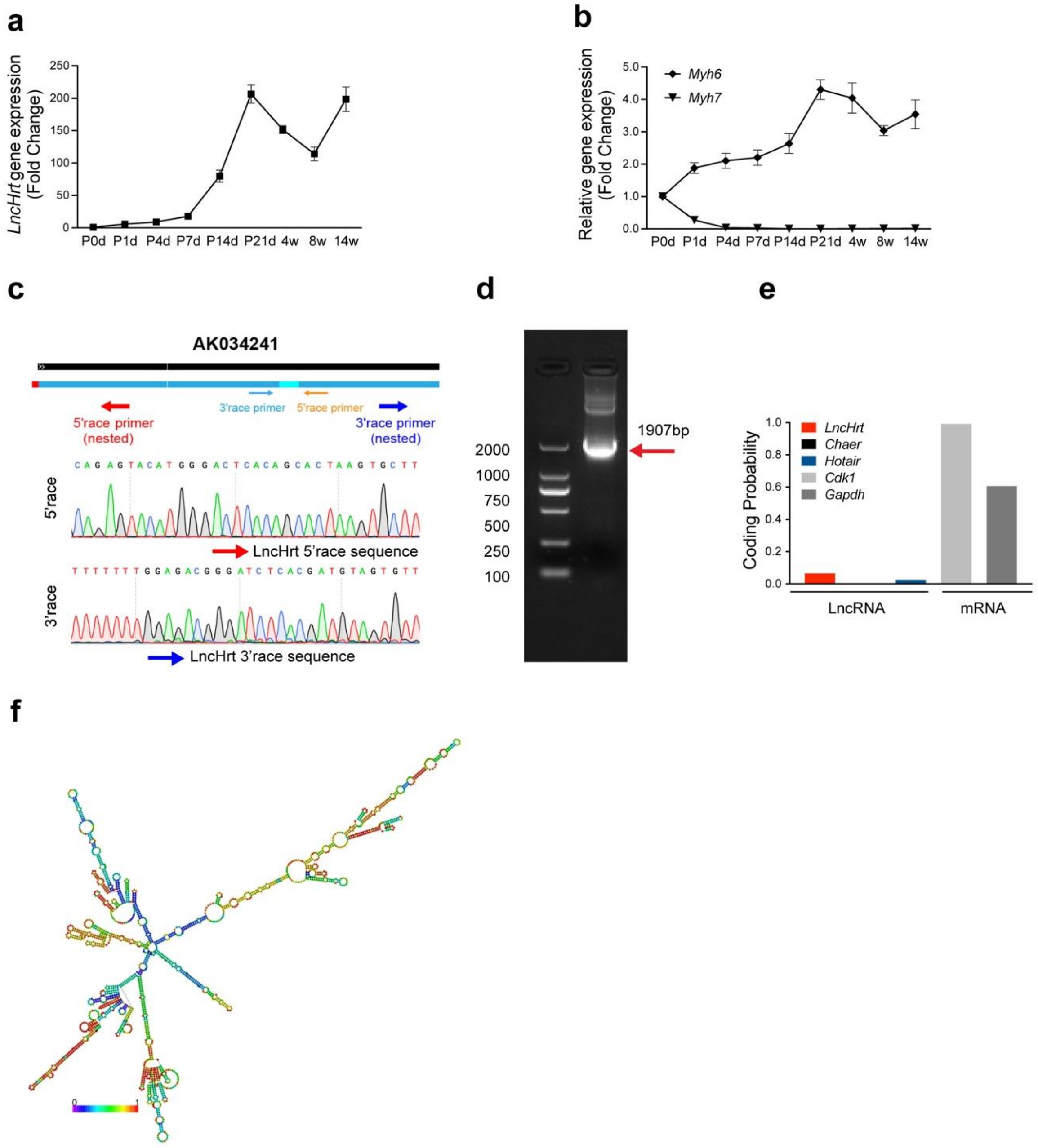

Supplementary Figure 1. *LncHrt* is a cardiac myocyte-enriched LncRNA down-regulated after myocardial infarction.

a. *LncHrt* gene expression in heart development, n=4 mice per time point.

b. Heart development marker *Myh6* and *Myh7* genes expression in the heart development, n=4 mice per time point.

c. Schematic diagram of *LncHrt* 5′ and 3′ rapid amplification of cDNA (complementary DNA) ends (RACE)（upper）. Sequencing of PCR products indicates the boundary between the universal anchor primer and *LncHrt* sequences (lower). The arrow indicates the *LncHrt* 5′ and 3′ cDNA ends sequence.

d. The agarose gel electrophoresis image of *LncHrt* RT-PCR products from mice heart tissue cDNA.

e. Coding probability of *LncHrt* predicted by CPAT. Protein coding mRNAs *Cdk1* and *Gapdh* serve as negative controls and reported LncRNAs *Chaer* and *Hotair* are positive controls.

f. Prediction of *LncHrt* secondary structure based on minimum free energy (MFE) and partition function by RegRNA Server with default settings.

Supplementary Figure 2

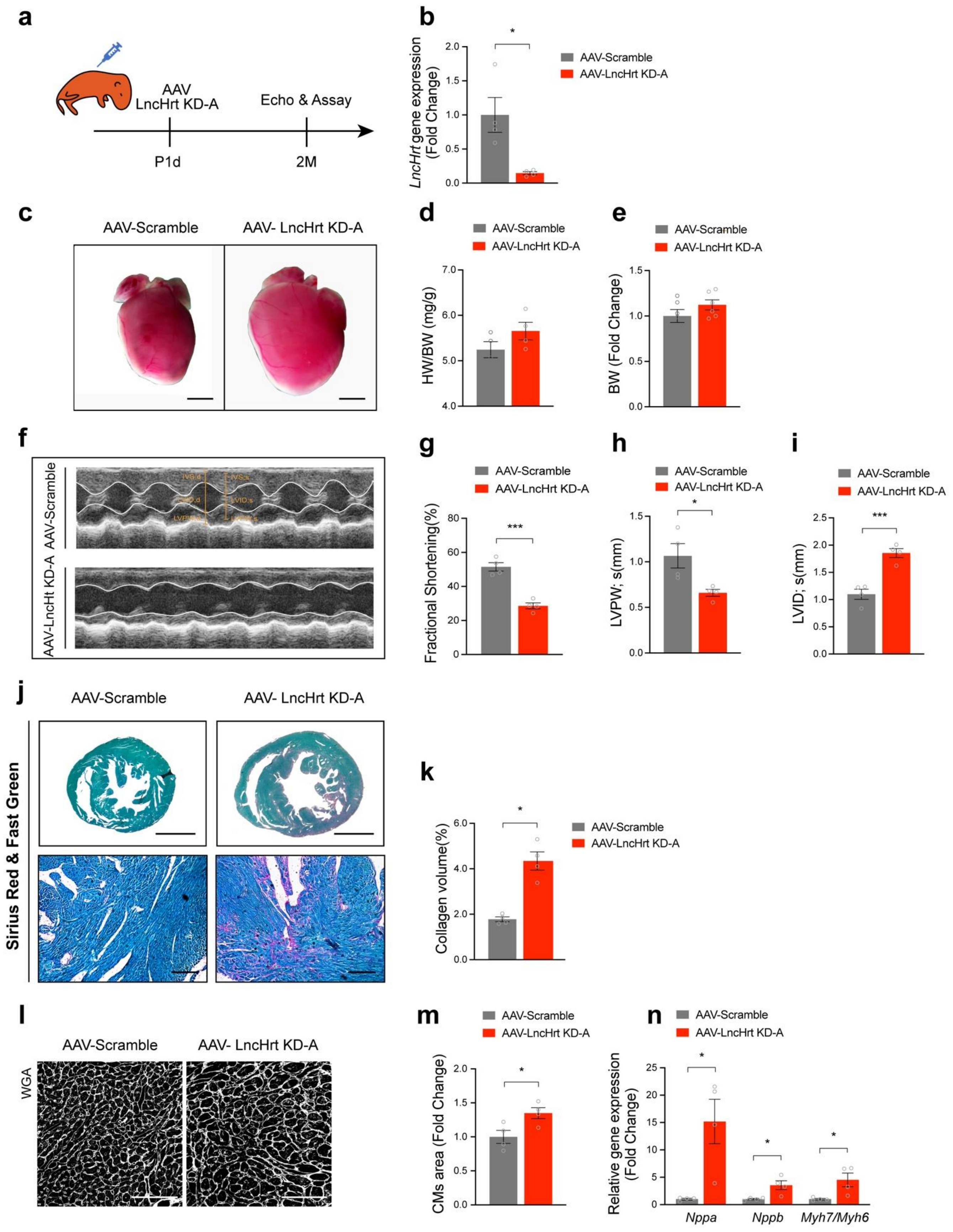

Supplementary Figure 2. Knockdown of *LncHrt* impairs cardiac homeostasis

a. Graphical representation showing the experimental procedures for *LncHrt* knockdown by AAV9-delivered *LncHrt*-shRNA at the site of A (KD-A) in mice.
b. qRT-PCR of *LncHrt* gene expression. *Rn18S* as a reference gene. Data are mean± s.e.m. * *P*<0.05 and ** P<0.01(versus AAV-Scramble); by Mann-Whitney test, n=4 mice per group.
c. Representative images of gross morphology of hearts from AAV-scramble and AAV-LncHrt KD-A. Scale bar =2mm.
d. Heart weight (HW) to body weight (BW) ratio in AAV-Scramble and AAV-LncHrt KD-A heart at 2~3 months. Data are mean±s.e.m. * P<0.05 and ** P<0.01(versus AAV-Scramble); by Student's t test, n=4 mice per group.
e. Body weight (BW) Fold Change in AAV-Scramble and AAV-LncHrt KD-A heart at 2 months. Data are mean±s.e.m. * P<0.05 and ** P<0.01(versus AAV-Scramble); by Student's t test, n=4 mice per group.
f. M-mode echocardiography of mice heart 2 month after AAV9-delivered LncHrt-shRNA injection.

g-i. Echocardiography analyses of cardiac function of FS% (g), LVPW (h) and. LVID (i) after AAV9 delivered LncHrt-shRNA injection at 2 months compared to the scramble group. FS, left ventricular fractional shortening. LVPWs, left ventricular posterior wall at end-systole. LVID,s, LV internal dimension at end-systole. Data are mean±s.e.m. * P<0.05 and ** P<0.01(versus AAV-Scramble); by Student's t test, n=4 mice per group.

j. Representative of low- (top) and high-magnification (bottom) of images of Sirius red & fast green staining of scramble and *LncHrt* knock-down heart sections, Scale bars, 2mm (up), 50μM (down).
k. Quantification of collagen volume of scramble and *LncHrt* knocking down hearts. Data are mean±s.e.m. * P<0.05 and ** P<0.01 (versus AAV-Scramble); by Student's t test, n=4 mice per group.
l. Representative pictures of cardiomyocytes stained with Wheat Germ Agglutinin (WGA). Scale bars= 100 μm.
m. Quantification of cardiomyocytes area of AAV-LncHrt KD-A compared to AAV-Scramble group. Data are mean±s.e.m. *** P<0.001 (versus AAV-Scramble); by Student's t test, n=4 mice hearts per group, measured ~400 cardiomyocytes per heart and calculated median value for each heart.
n. Gene expression of cardiac disease markers in scramble and *LncHrt* knocking down hearts. *Rn18S* as a reference gene. Data are mean±s.e.m. * P<0.05 and ** P<0.01(versus AAV-Scramble); by Mann-Whitney test, n=4 mice per group.

Supplementary Figure 3

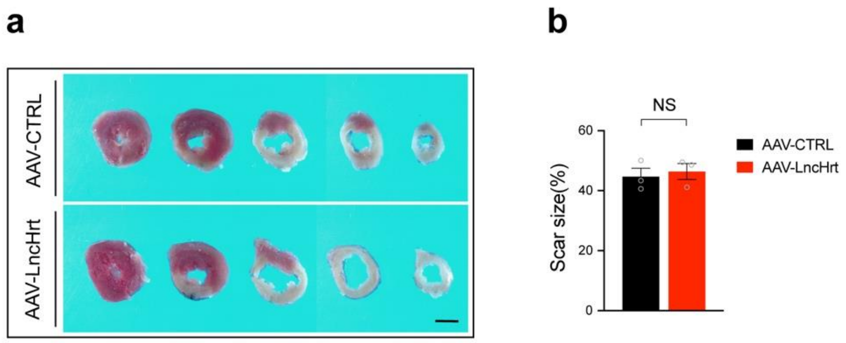

Supplementary Figure 3. AAV9-delivered *LncHrt* overexpression protects the heart from myocardial infarction.

a. TTC viability staining in myocardial necrosis (white) in both AAV-CTRL and AAV-LncHrt injected mouse hearts at 1 day post-MI. Scale bar=2mm.

b. Quantification of infarct size in both AAV-CTRL and AAV-LncHrt injected mice at 1 day post-MI. Data are mean±s.e.m. * $P<0.05$ and ** $P<0.01$ (versus AAV-CTRL); by Student's t test, n=3 mice per group.

Supplementary Figure 4

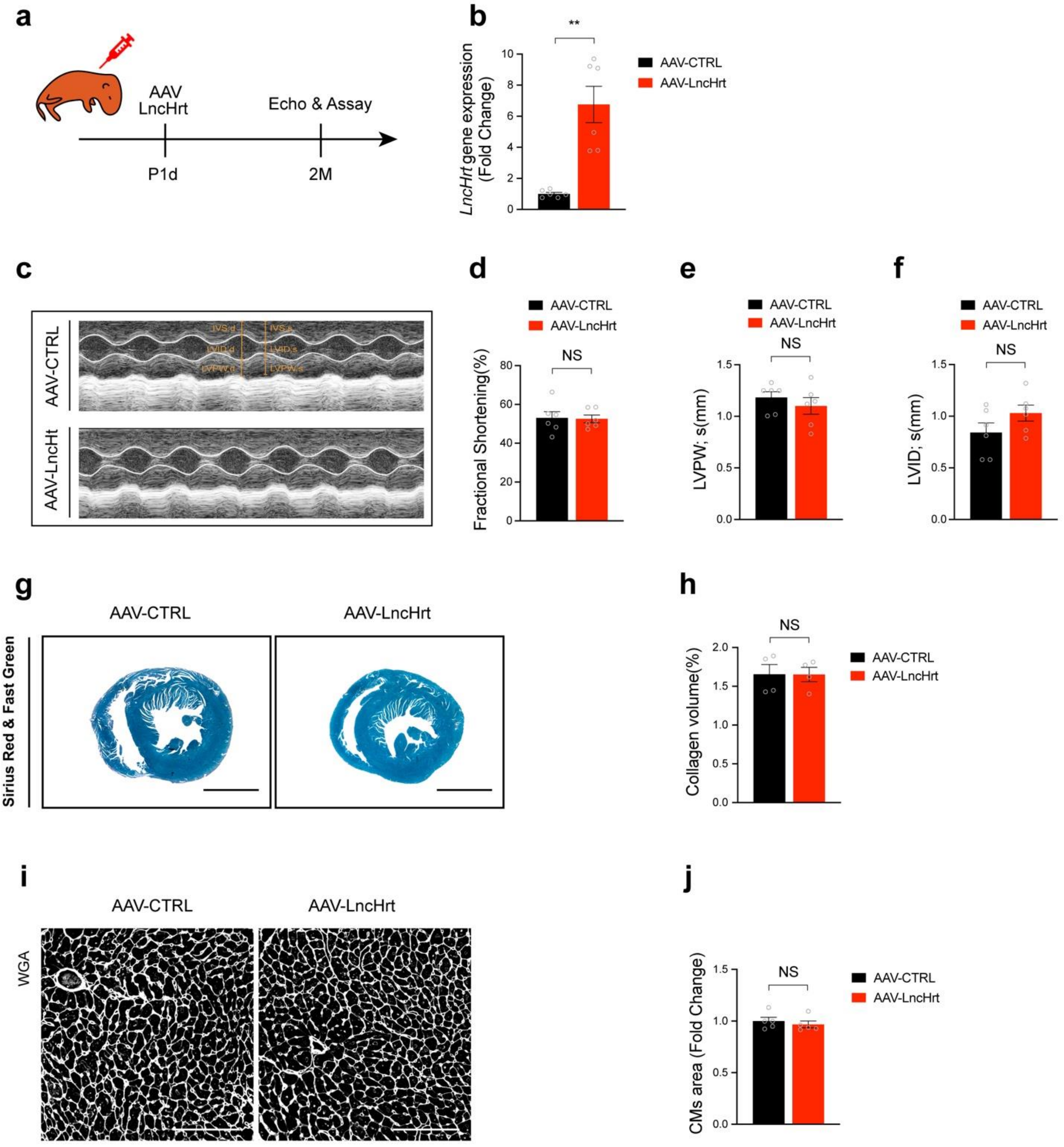

Supplementary Figure 4. *LncHrt* does not affect cardiac function and morphology in the healthy heart.

a. Graphical representation showing the experimental procedures for AAV9-delivered *LncHrt* overexpression in healthy heart.

b. qRT-PCR of *LncHrt* gene expression. *Rn18S* as a reference gene. Data are mean± s.e.m. * P<0.05 and ** P<0.01 (versus AAV-CTRL); by Mann-Whitney test, n=6 mice per group.

c. M-mode echocardiography of mouse hearts 2 months after AAV9-delivered *LncHrt* injection.

d-f. Echocardiography analyses of cardiac function of FS% (d), LVPW (e) and. LVID (f) after. AAV9-delivered *LncHrt* injection 2 months compared to CTRL group. FS, left ventricular fractional shortening. LVPWs, left ventricular posterior wall at end-systole. LVID,s, LV internal dimension at end-systole. Data are mean±s.e.m. * P<0.05 and ** P<0.01(AAV-CTRL); by Student's t test, n=6 mice per group.

g. Representative images of Sirius red & fast green staining of CTRL and *LncHrt* overexpression heart sections, Scale bars= 2mm.

h. Quantification of collagen volume of CTRL and *LncHrt* overexpression hearts. Data are mean±s.e.m. * P<0.05 and ** P<0.01 (versus AAV-CTRL); by Student's t test, n=4 mice.

i. Representative pictures of cardiomyocytes stained with WGA. Scale bars= 100 μm.

j. Quantification of cardiomyocyte area Fold Change. Data are mean±s.e.m. *P<0.05 (versus AAV-CTRL); by Student's t test, n=5 hearts for each group, about 200 cardiomyocytes measured per heart for each group.

Supplementary Figure 5

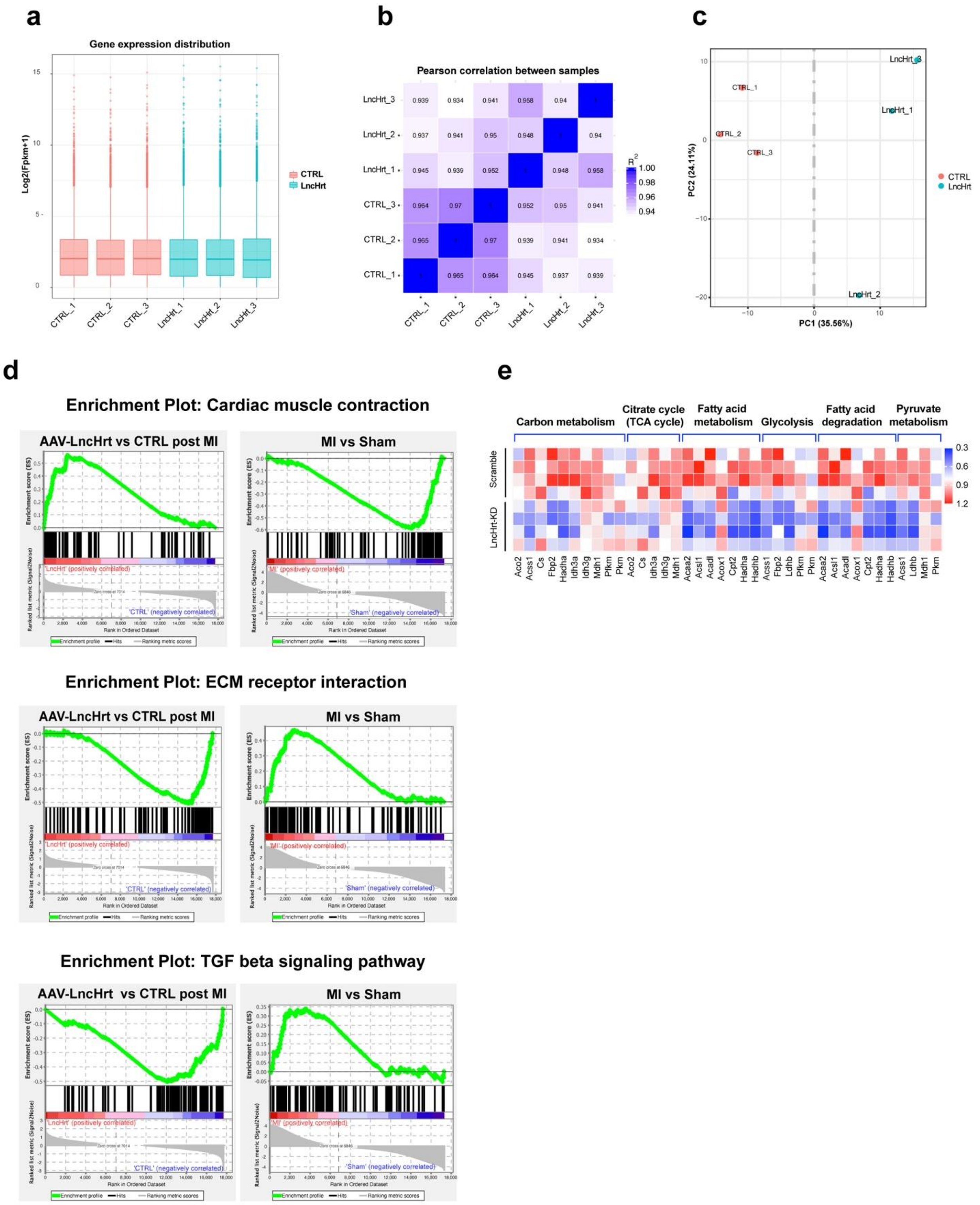

Supplementary Figure 5. Cardiac *LncHrt* overexpression rescues the transcriptome from myocardial infarction.

a. Boxplot showing gene expression distribution (Log2(Fpkm+1)) of indicated samples.

b. Hierarchical clustering showing the sample distance with the whole transcriptome profile.

c. PCA analysis showing the consistence between the replicates.

d. GSEA analysis of cardiac repair related enrichment plots which were rescued by *LncHrt* against MI.

e. Heatmap of metabolic related genes validation by qRT-PCR after inhibited *LncHrt* expression

Supplementary Figure 6

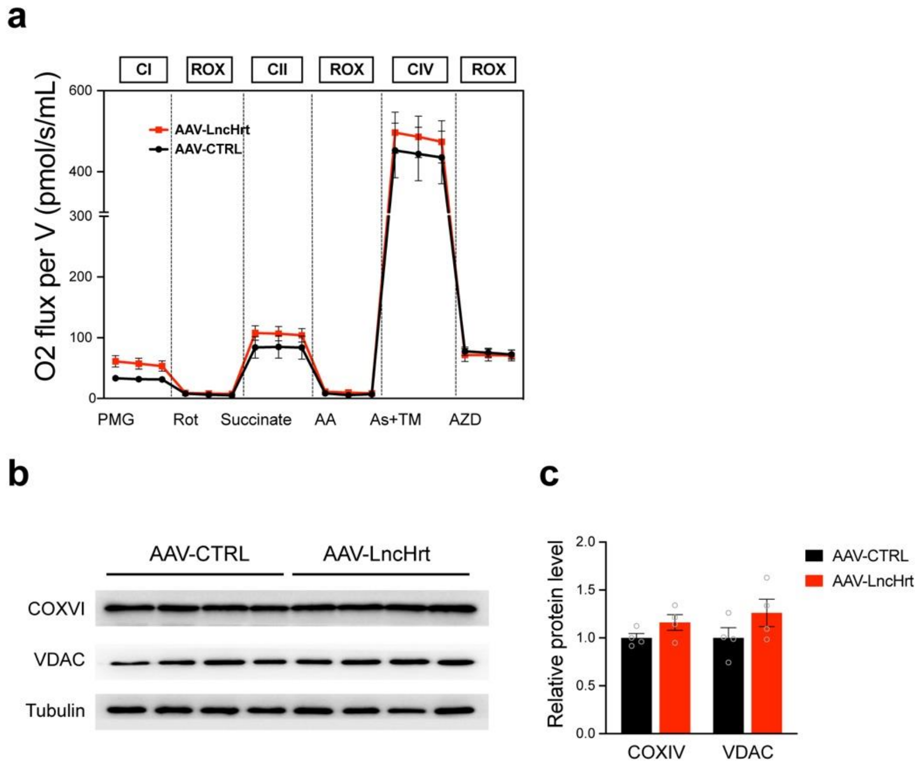

Supplementary Figure 6. *LncHrt* improves cardiac metabolic homeostasis after myocardial infarction.

a. Representative respiratory experiment of mitochondrial oxidative phosphorylation (OXPHOS) capacity of complex in electron transfer (ET) state by using application of substrate uncoupler inhibitor titration (SUIT) protocols to interrogate sequentially different substrate and coupling states using saponin permeabilized myofibers.

b. Western blot of mitochondrion content marker protein.

c. Quantification of western blot band density using image J. Data are mean±s.e.m. * $P<0.05$ and ** $P<0.01$(versus AAV-CTRL); by Student’s t test, n=4 mice per group.

Supplementary Figure 7

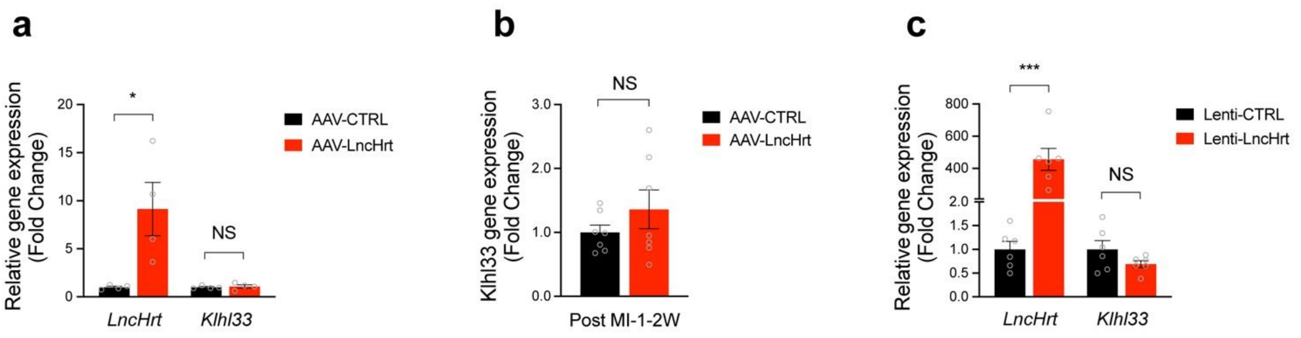

Supplementary Figure 7. *LncHrt* activates the LKB1-AMPK signaling pathway by interacting with SIRT2

a. qRT-PCR of *LncHrt* and its neighbor gene *Klhl33* expression in baseline. *Rn18S* as a reference gene. Data are mean± s.e.m. * P<0.05 and ** P<0.01(versus AAV-CTRL); by Student's t'test, n= 4 mice.
b. qRT-PCR of *Klhl33* gene expression post MI. *Rn18S* as a reference gene. Data are mean± s.e.m. * P<0.05 and ** P<0.01(versus AAV-CTRL); by Mann-Whitney test, n= 4 mice.
c. qRT-PCR of *LncHrt* and *Klhl33* gene expression in cardiomyocytes. *Rn18S* as a reference gene. Data are mean± s.e.m. * P<0.05 and ** P<0.01(versus lenti-CTRL); by Student's t'test, n= 4 mice.

Supplementary Figure 8

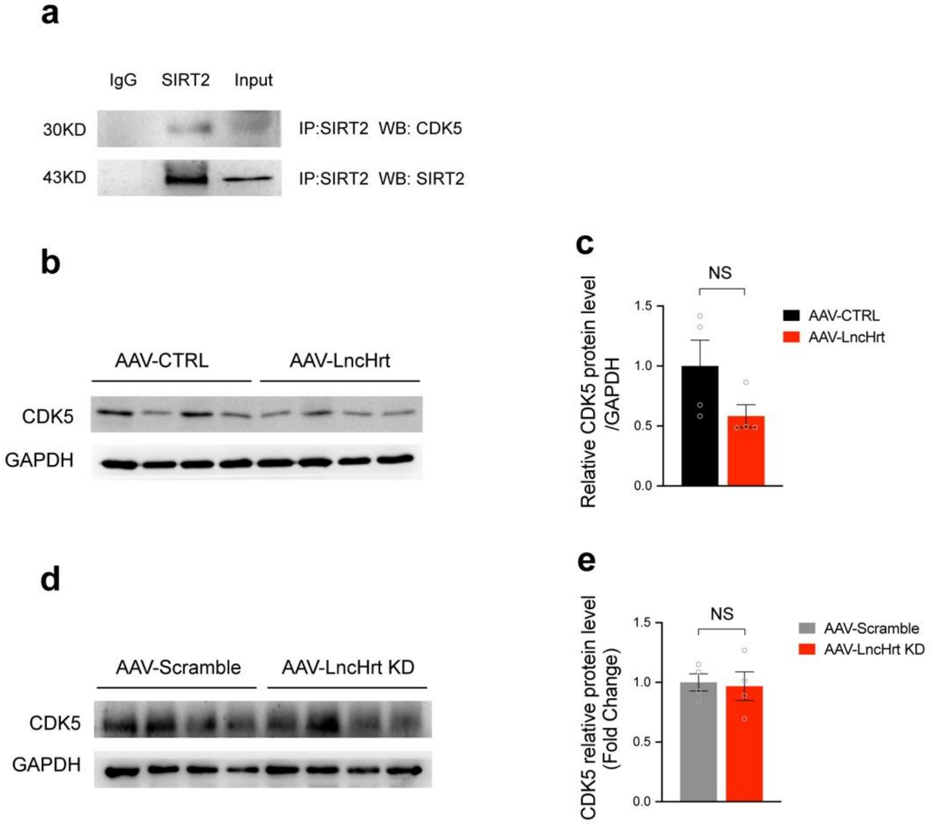

Supplementary Figure 8. *LncHrt* preserved SIRT2 activity by interfering CDK5 inhibition on SIRT2

a. Western blot of Co-immunoprecipitated CDK5 and SIRT2 using anti-SIRT2 antibody with negative control IgG antibody in heart tissue lysates.
b. Western blot of CDK5 protein in AAV-LncHrt injected heart tissues post MI.
c. Quantification of protein expression of CDK5. Data are mean± s.e.m. * $P<0.05$ and ** $P<0.01$(versus AAV-CTRL); by Student's t'test, n=4 mice.
d. Western blot of CDK5 and SIRT2 protein in AAV-LncHrt KD heart tissues.
e. Quantification of protein expression of CDK5 and SIRT2. Data are mean± s.e.m. * $P<0.05$ and ** $P<0.01$(versus AAV-Scramble); by Student's t'test, n=4 mice.

Supplementary Figure 9

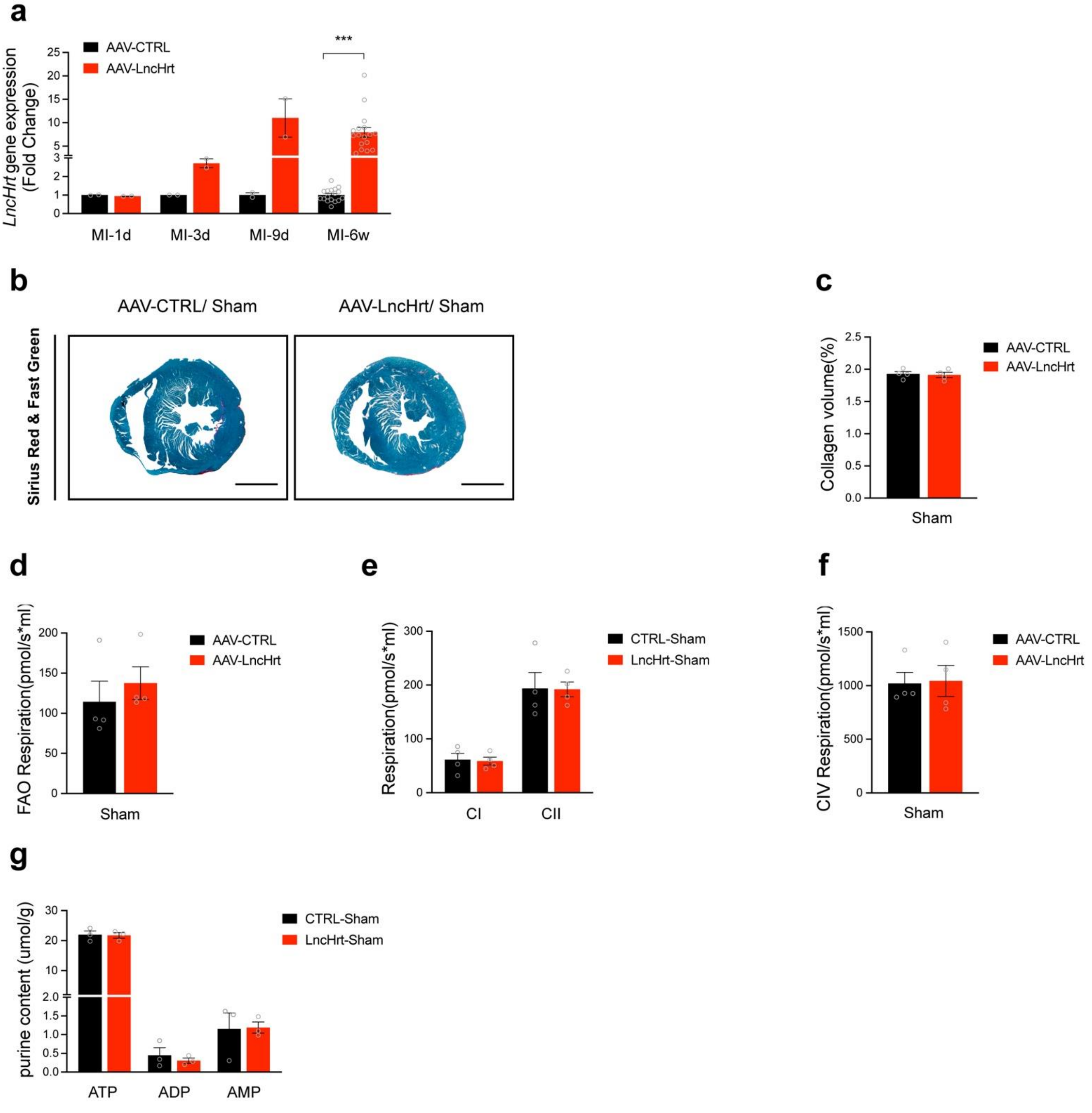

Supplementary Figure 9. Therapeutic potential of *LncHrt* in infarcted hearts

a. qRT-PCR of *LncHrt* gene expression at 1 day, 3 day, 9 day and 6 weeks after MI. *Rn18S* as a reference gene. Data are mean±s.e.m. *** P<0.001 (versus AAV-CTRL); by Mann-Whitney test, n=2 mice per group in MI-1d, -3d,-9d and n= 17 mice per group in MI-6W.
b. Representative images of Sirius red & fast green staining of CTRL and *LncHrt* overexpressed sham heart sections, Scale bars= 2mm.
c. Quantification of collagen volume of CTRL and *LncHrt* overexpressed sham hearts. Data are mean±s.e.m. * P<0.05 and ** P<0.01 (versus AAV-CTRL); by Student's t test, n=4 mice.
d. Fatty acid oxidation (FAO) in sham groups, as measured by oxygen consumption with octanoylcarnitine

corrected to corresponding residual oxygen consumption (ROX). Data are mean±s.e.m. * $P<0.05$ and ** $P<0.01$(versus AAV-CTRL); by Student’s t test, n=4 mice per group.

e. Mitochondrial respiration of complex I (CI) and complex II (CII) in sham groups. The oxygen consumption rate (OCR) of complex I showed oxygen consumption with Pyruvate, Malate, and Glutamate (PMG) titrations minus octanoylcarnitine titrations, while complex II respiration rate was corrected to corresponding residual oxygen consumption (ROX). Data are mean±s.e.m. * $P<0.05$ and ** $P<0.01$ (versus CTRL-Sham); by Student’s t test, n=4 mice per group.
f. Mitochondrial oxidative phosphorylation (OXPHOS) capacity of complex IV (CIV) in sham groups as illustrated by oxygen consumption rate (OCR) corrected to the corresponding residual oxygen consumption (ROX). Data are mean±s.e.m. * $P<0.05$ and ** $P<0.01$(versus AAV-CTRL); by Student’s t test, n=4 mice per group.
g. Purine content in heart tissues normalize to protein levels in sham groups. Data are mean±s.e.m. * $P<0.05$, ** $P<0.01$ and *** $P<0.001$ (versus CTRL-Sham); by Student’s t test, n=3 mice per group.
