# Supplementary material for: LncRNA LncHrt preserves cardiac metabolic homeostasis and heart function by modulating the LKB1-AMPK signaling pathway
Source: Basic Res Cardiol. 2021 Aug 11;116(1):48. doi: 10.1007/s00395-021-00887-3 (PMC8357683; doi:10.1007/s00395-021-00887-3)
Supplement: Supplementary file 2 — Supplementary file2 (PDF 323 KB) [file 395_2021_887_MOESM2_ESM.pdf]

Supplementary Table 1. Down-regulated 36 LncRNAs between MI-3d and MI-14d.

Probe Name	Fold change (MI3d vs sham)	Fold change (MI14d vs sham)	GenBank Accession /Ensemble ID	Gene Name
**A_55_P2197134**	**-22.882**	**-3.933**	**AK034241**	**RIKEN cDNA A930018M24 gene**
**A_55_P2199382**	**-14.429**	**-6.463**	**NR_045336**	**predicted gene 10635**
**A_66_P123980**	**-12.639**	**-5.028**	**XR_373578**	**RIKEN cDNA 2310015K22 gene**
**A_55_P2042783**	**-11.092**	**-3.089**	**NR_131053**	**RIKEN cDNA 2310075C17 gene**
**A_55_P2272032**	**-10.138**	**-2.347**	**XR_386048**	**RIKEN cDNA 9130001E16 gene**
A_55_P2301713	-6.806	-2.566	AK034922	RIKEN cDNA 9430062P05 gene
A_66_P122719	-6.753	-2.866	AK081938	
A_66_P125223	-6.176	-2.661	NR_033261	proline rich 33
A_52_P770640	-5.685	-3.536	AK147615	uncharacterized LOC100047044
A_55_P1963724	-5.661	-2.591	ENSMUST00000122117	
A_51_P274137	-5.215	-2.232	NR_028122	RIKEN cDNA 2310001K24 gene
A_66_P109243	-5.205	-2.392	NR_046023	predicted gene 6416
A_66_P119350	-5.127	-2.066	NR_040293	RIKEN cDNA 2310040G24 gene
A_55_P2407367	-5.111	-3.922	XR_386048	RIKEN cDNA 9130001E16 gene
A_55_P2292881	-4.991	-3.545	NR_046027	predicted gene, 20757
A_55_P2401009	-4.966	-2.265	AK020961	RIKEN cDNA B230110C06 gene
A_55_P1986341	-4.695	-2.280	NR_002858	predicted gene 4956
A_51_P477285	-4.613	-2.009	NR_015572	RIKEN cDNA 1810014B01 gene
A_55_P2179060	-4.482	-2.874	XR_001784803	predicted gene 3510
A_55_P2292933	-4.477	-2.394	AK053225	RIKEN cDNA E030046B03 gene
A_66_P125828	-4.368	-2.198	ENSMUST00000053717	
A_52_P305053	-4.238	-2.228	NR_030695	RIKEN cDNA B330016D10 gene
A_55_P2166799	-3.991	-2.069	ENSMUST00000175829	
A_55_P2263342	-3.758	-2.290	NR_015613	RIKEN cDNA 1700029M20 gene
A_55_P2067451	-3.449	-2.161	M73262	
A_66_P132971	-3.262	-2.543	AK015234	RIKEN cDNA 4930429E23 gene
A_55_P2217851	-3.245	-2.041	NR_073360	RIKEN cDNA 4930453L07 gene
A_55_P2278551	-3.174	-2.252	BB242157	RIKEN cDNA 1700037F03 gene
A_55_P2054042	-3.152	-2.687	NR_033551	predicted gene 12359
A_66_P130759	-3.048	-2.170	NR_110333	RIKEN cDNA 4631405J19 gene
A_55_P2060474	-2.991	-2.739	NR_045335	predicted gene 13582
A_66_P107258	-2.884	-2.181	AK050354	predicted gene 14168
A_66_P108462	-2.724	-2.317	NR_046013	RIKEN cDNA D830013O20 gene
A_55_P2199437	-2.594	-2.246	NR_045873	predicted gene 11201
A_55_P2308283	-2.444	-2.554	NR_045686	predicted gene 16062
A_55_P2075248	-2.276	-2.006	AK134269	predicted gene 10501

Supplementary Table 2. Patient characteristics

Patients	Sex	Age(years)	Pathology
CTRL-1	M	15	None(Donor)
CTRL-2	M	30	None(Donor)
DCM-1	M	49	DCM（arrhythmia）
DCM-2	M	68	DCM（atrial fibrillation, AF）
DCM-3	M	57	DCM（atrial fibrillation, AF）
DCM-4	F	52	DCM（diabetes）
DCM-5	M	62	DCM
DCM-6	M	55	DCM

Supplementary Table 3. Echocardiography examination of cardiac function of AAV-LncHrt KD or AAV-Scramble injected heart at 2 -3 months in baseline.

	2 Months at baseline			
	AAV-Scramble (n=7)	AAV-LncHrt KD (n=7)	AAV- Scramble (n=4)	AAV-LncHrt KD-A (n=4)
IVS;d (mm)	0.883±0.090	0.903±0.129	0.984±0.132	0.856±0.233
IVS;s (mm)	1.513±0.109	1.443±0.154	1.576±0.095	1.172±0.117**
LVID;d (mm)	2.589±0.192	3.296±0.169***	2.263±0.307	2.595±0.168
LVID;s (mm)	1.077±0.321	2.049±0.217***	1.098±0.185	1.855±0.165***
LVPW;d (mm)	0.687±0.091	0.687±0.115	0.927±0.213	0.718±0.104
LVPW;s (mm)	1.294±0.148	1.064±0.198*	1.066±0.267	0.660±0.076*
EF (%)	88.869±6.769	68.705±7.681***	84.572±4.112	57.078±5.470***
FS (%)	58.899±9.990	37.796±6.310***	51.539±4.958	28.582±3.525***
LV Mass (mg)	58.017±11.212	85.018±10.263***	65.277±18.283	58.109±13.352
LV Mass (Corrected, mg)	46.414±8.969	68.014±8.210***	52.221±14.626	46.488±10.682
LV Vol;d (uL)	24.545±4.539	44.178±5.485***	17.835±6.096	24.623±4.004
LV Vol;s (uL)	2.923±2.063	13.769±3.662***	2.768±1.098	10.611±2.304***

Wild type C57BL/6 mice were randomly subjected to subcutaneous injection of Adenovirus associated virus of AAV-LncHrt KD of two different sites or AAV-Scramble. Echocardiography analyses of cardiac function were performed at 2 Months in baseline. N of each group was indicated. *: P<0.05; **: P<0.01 vs. control group. IVS;d: Interventricular septal thickness at diastole; IVS;s Interventricular septal thickness at systole; LVID;d: Left ventricular end diastolic internal dimension; LVID;s: Left ventricular end systolic internal dimension; LVPW;d: Left ventricular end diastolic posterior wall dimension; LVPW;s: Left ventricular end systolic posterior wall dimension; EF: Ejection fraction; FS: Fractional shortening; LV Vol;d: Left ventricular end diastolic volume. LV Vol;s: Left ventricular end systolic volume.

Supplementary Table 4. Echocardiography examination of cardiac function of AAV-LncHrt or AAV-CTRL injected heart at multiple time points post-MI.

	Pre-MI		1 months post-MI		2 months post-MI	
	AAV-CTRL (n=14)	AAV-LncHrt (n=16)	AAV-CTRL (n=14)	AAV-LncHrt (n=16)	AAV-CTRL (n=14)	AAV-LncHrt (n=16)
**IVS;d (mm)**	1.027±0.166	1.022±0.105	0.618±0.322	0.970±0.228**	0.610±0.453	1.104±0.277**
**IVS;s (mm)**	1.503±0.179	1.567±0.158	0.761±0.506	1.300±0.336**	0.724±0.613	1.419±0.327**
**LVID;d (mm)**	2.002±0.226	2.199±0.262	3.978±0.628	3.608±0.716	4.027±0.622	3.594±0.775
**LVID;s (mm)**	0.961±0.230	1.051±0.239	3.324±0.604	2.746±0.766*	3.380±0.705	2.683±0.790*
**LVPW;d (mm)**	0.810±0.144	0.751±0.148	0.547±0.195	0.736±0.265*	0.554±0.208	0.706±0.210
**LVPW;s (mm)**	1.049±0.160	1.054±0.178	0.691±0.242	0.903±0.386	0.640±0.288	0.917±0.300*
**EF (%)**	85.222±5.507	85.015±5.584	34.527±11.899	49.756±12.333**	34.684±12.614	51.970±13.457**
**FS (%)**	52.423±6.982	52.599±7.637	16.478±6.482	25.041±7.518**	16.594±6.897	26.547±8.544**
**LV Mass (mg)**	51.651±11.418	55.874±15.008	78.723±28.779	107.967±24.471**	80.809±43.983	118.798±24.066**
**LV Mass(Corrected, mg)**	41.321±9.134	44.699±12.006	62.978±23.023	86.374±19.577**	64.647±35.186	95.038±19.253**
**LV Vol;d (uL)**	13.041±4.014	16.577±4.927*	71.506±25.364	57.856±25.717	73.496±25.876	57.882±26.495
**LV Vol;s (uL)**	2.071±1.450	2.594±1.336	47.084±20.444	31.617±19.397*	49.756±23.724	30.316±18.180*

Wild type C57BL/6 mice were randomly subjected to subcutaneous injection of AAV-LncHrt or AAV-CTRL then treated with myocardium infarction (MI) surgery, respectively. Echocardiography analyses of cardiac function were performed at multiple time points post-MI surgery. N of each group was indicated. *: $P<0.05$; **: $P<0.01$ vs. control group. IVS;d: Interventricular septal thickness at diastole; IVS;s Interventricular septal thickness at systole; LVID;d: Left ventricular end diastolic internal dimension; LVID;s: Left ventricular end systolic internal dimension; LVPW;d: Left ventricular end diastolic posterior wall dimension; LVPW;s: Left ventricular end systolic posterior wall dimension; EF: Ejection fraction; FS: Fractional shortening; LV Vol;d: Left ventricular end diastolic volume. LV Vol;s: Left ventricular end systolic volume.

Supplementary Table 5. Echocardiography examination of cardiac function of AAV-LncHrt or AAV-CTRL injected heart at 2 months of healthy heart.

	2 Months of sham heart	
	**AAV-CTRL** (n=6)	**AAV-LncHrt** (n=6)
**IVS;d (mm)**	1.173±0.147	1.066±0.141
**IVS;s (mm)**	1.639±0.138	1.579±0.174
**LVID;d (mm)**	1.928±0.202	2.158±0.207
**LVID;s (mm)**	0.842±0.229	1.030±0.190
**LVPW;d (mm)**	0.766±0.190	0.772±0.110
**LVPW;s (mm)**	1.183±0.136	1.101±0.199
**EF (%)**	85.603±6.085	85.500±4.009
**FS (%)**	53.040±7.922	52.565±4.886
**LV Mass (mg)**	55.717±12.079	56.610±7.437
**LV Mass (Corrected, mg)**	44.574±9.663	45.288±5.949
**LV Vol;d (uL)**	12.956±3.054	15.653±3.863
**LV Vol;s (uL)**	2.000±1.149	2.369±1.157

Wild type C57BL/6 mice were randomly subjected to subcutaneous injection of Adenovirus associated virus of AAV-LncHrt or AAV-CTRL. Echocardiography analyses of cardiac function were performed at 2 Months of healthy heart. N of each group was indicated. *: $P<0.05$; **: $P<0.01$ vs. control group. IVS;d: Interventricular septal thickness at diastole; IVS;s Interventricular septal thickness at systole; LVID;d: Left ventricular end diastolic internal dimension; LVID;s: Left ventricular end systolic internal dimension; LVPW;d: Left ventricular end diastolic posterior wall dimension; LVPW;s: Left ventricular end systolic posterior wall dimension; EF: Ejection fraction; FS: Fractional shortening; LV Vol;d: Left ventricular end diastolic volume. LV Vol;s: Left ventricular end systolic volume.

Supplementary Table 6. Mass spectrometry results of proteins associated with LncHrt. (Score>1, Unique peptides >1 and PSMs>1)

Gene name	Accession	Score	Coverage	Unique Peptides	Peptides	PSMs	MW [kDa]
Mapk9	Q9WTU6-2	10.83	16.01	5	6	6	44.0
Map4k4	B7ZNR9	1.92	2.81	3	4	4	137.8
Picalm	Q7M6Y3-2	12.03	20.27	10	10	11	64.6
Pdlim5	D9J2Z9	1.97	11.41	4	4	5	52.4
Mbnl2	Q8C181-3	1.90	5.1	2	2	2	35.9
Arhgef1	Q61210	12.73	17.07	11	11	12	102.7
Flna	B7FAV1	9.06	5.46	11	11	11	274.5
Septin-2	P42208	5.30	16.34	5	5	6	41.5
Ap2b1	Q9DBG3	25.47	24.97	9	21	23	104.5
Strip1	Q8C079-3	6.79	8.01	5	5	5	86.8
Rps2-ps6	A0A140T8L5	3.50	13.57	3	3	3	24.2
Stat1	Q99K94	39.88	33.29	21	21	27	83.1
Gm5580	A0A0N4SVP8	17.34	17.52	5	9	10	46.9
P4ha2	Q60716-2	13.60	25.23	9	9	9	60.8
Ctnnb1	Q02248	3.94	6.27	5	5	5	85.4
Atp5c1	A2AKV1	2.22	13.64	2	2	2	16.8
Ube3a	O08759-2	1.63	11.66	9	9	9	97.5
Rpl18a	A0A1D5RME4	2.73	12.20	2	2	2	14.4
Psmd4	O35226	1.69	9.84	3	3	3	40.7
Psmc3	A2AGN7	13.56	30.50	9	9	10	44.6
P3h1	Q3V1T4-3	6.69	7.14	4	4	4	64.1
**Sirt2**	**A0A140LHL5**	**5.16**	**11.40**	**4**	**4**	**4**	**39.4**
Myl12b	Q3THE2	3.54	15.70	2	2	2	19.8
Adsl	P54822	2.88	8.06	4	4	4	54.8
Cpne1	V9GXM6	2.38	12.69	2	2	2	22.0
Mms19	Q9D071-3	1.84	2.63	3	3	3	108.2
Txnrd1	H3BJS1	1.77	11.89	2	2	2	20.5
Tfg	B8JJG9	1.71	30.34	2	2	2	10.2
Mpp1	A2AN84	1.61	10.40	4	4	4	50.5
Myo1c	Q9WTI7-2	34.07	29.38	25	25	25	118.1
Cul5	E9Q6Z0	12.28	14.90	11	11	11	75.9
Parp9	Q8CAS9	5.17	17.90	11	11	11	96.6
Ube2m	G5E919	5.15	33.72	3	3	3	10.1
Hnrnpf	Q9Z2X1-2	4.35	8.61	2	3	4	43.7
Cdc23	Q8BGZ4-2	3.29	3.97	2	2	2	55.8
Nek7	Q3TN15	2.60	8.96	2	2	2	22.7
Elmo2	Q8BHL5-2	2.21	2.36	2	2	2	82.5
Cbl	A0A0U1RP47	2.09	3.24	2	2	2	98.3
Uchl5	Q9WUP7-2	1.84	13.41	5	5	5	37.5
Tbc1d17	Q8BYH7	1.82	6.20	4	4	4	72.8
Dhx15	A0A0G2JG10	1.76	7.40	4	4	4	68.5
Cops4	O88544	1.66	5.91	2	2	2	46.3

Gm49369	A0A1B0GT66	1.66	13.19	3	3	3	29.4
Ddx3y	Q62095	34.19	29.33	2	17	21	73.4
Dync1li2	Q6PDL0	7.25	20.93	8	8	9	54.2
Vav1	Q8VDU4	5.88	3.47	3	3	3	93.8
Psmd8	Q3TG45	5.62	13.44	3	3	3	28.5
Mnda	P0DOV1	4.30	15.06	3	6	7	46.9
Capza2	A0A0N4SVM0	4.17	15.38	2	2	2	19.5
Vps4a	Q8VEJ9	3.93	4.58	2	2	2	48.9
Vps51	Q3UVL4	3.64	5.63	4	4	4	86.1
Impa1	O55023	3.64	14.08	4	4	4	30.4
Fxr2	Q6P5B5	2.20	7.86	2	4	4	74.2
Cope	D3Z315	2.01	14.54	3	3	3	25.4
Sgpl1	D6REF7	1.97	3.90	2	2	2	54.7
Vac14	A0A1D5RLY2	1.96	8.89	4	4	4	51.5
Fam129a	E9PYV4	1.87	3.60	2	2	2	66.8
Exoc5	Q3TPX4	1.79	6.92	4	4	4	81.7
Ccdc93	E9QAD4	8.93	12.74	8	8	8	72.4
Hook3	Q8BUK6	5.46	6.96	5	5	5	83.2
Tnpo2	Q99LG2	4.82	5.41	2	4	4	100.4
Cttn	Q921L6	4.20	3.93	2	2	2	57.1
Syk	P48025	4.10	17.81	8	8	9	71.3
Myo1d	Q5SYD0	3.83	7.26	7	7	7	116.0
Cryab	A0A1L1SRG5	3.61	30.77	4	4	4	15.4
Serpinb6b	F7B9A0	3.37	15.24	3	3	3	23.1
Nnmt	O55239	2.73	8.33	2	2	3	29.6
Txnrd1	Q9JMH6-2	2.33	4.21	2	2	2	54.5
Hip1	A0A0J9YUA3	1.91	2.91	2	2	2	68.1
Ppcs	Q8VDG5	1.68	6.43	2	2	2	33.8
Pus10	Q9D3U0	3.62	6.83	3	4	4	59.7
Ist1	Q9CX00	2.37	5.25	2	2	2	39.4

Supplementary Table 7. Echocardiography examination of cardiac function of AAV9-LncHrt or AAV-CTRL intra-myocardial injected heart at multiple time points post-MI.

	1 day post-MI		1 Week post-MI		2 Weeks post-MI		4 Weeks post-MI		6 Weeks post-MI	
	AAV-CTRL (n=17)	AAV-LncHrt (n=17)	AAV-CTRL (n=17)	AAV-CTRL (n=17)	AAV-CTRL (n=17)	AAV-LncHrt (n=17)	AAV-CTRL (n=17)	AAV-LncHrt (n=17)	AAV-CTRL (n=17)	AAV-LncHrt (n=17)
**IVS;d (mm)**	0.862±0.188	0.861±0.159	0.769±0.177	0.784±0.210	0.578±0.234	0.683±0.203	0.661±0.183	0.83±0.182*	0.666±0.264	1.005±0.127***
**IVS;s (mm)**	1.093±0.251	1.056±0.198	1.030±0.266	0.963±0.268	0.751±0.377	0.989±0.33	0.854±0.324	1.104±0.249*	0.885±0.357	1.426±0.199***
**LVID;d (mm)**	3.792±0.270	4.009±0.596	4.785±0.476	4.385±0.390	4.850±0.376	4.644±0.351	4.907±0.441	4.298±0.643**	5.026±1.236	4.749±0.454
**LVID;s (mm)**	3.062±0.196	3.271±0.528	3.984±0.489	3.685±0.385	4.200±0.468	3.802±0.510*	4.205±0.558	3.412±0.664***	4.505±0.992	3.685±0.505**
**LVPW;d (mm)**	0.628±0.143	0.628±0.129	0.591±0.128	0.661±0.182	0.538±0.143	0.615±0.199	0.892±1.117	0.700±0.107	0.828±1.231	0.759±0.138
**LVPW;s (mm)**	0.800±0.213	0.811±0.205	0.677±0.156	0.855±0.274*	0.709±0.247	0.869±0.247	0.985±0.777	0.952±0.167	0.867±0.997	1.059±0.126
**EF (%)**	39.776±7.628	38.612±6.586	35.066±6.859	33.644±7.499	28.524±9.854	37.601±11.427*	30.457±9.91	42.661±8.521***	25.859±9.62	45.071±8.808***
**FS (%)**	19.104±4.335	18.486±3.630	16.834±3.636	15.975±3.999	13.506±5.215	18.389±6.536*	14.515±5.235	20.926±4.65***	12.224±4.988	22.573±5.166***
**LV Mass (mg)**	98.350±20.545	107.142±21.598	128.899±25.384	119.679±34.376	104.46±29.410	115.931±24.162	124.475±27.903	124.573±25.358	133.264±39.5	177.74±22.629***
**LV Mass (Corrected, mg)**	78.680±16.436	85.713±17.278	103.119±20.307	95.743±27.501	83.568±23.528	92.745±19.33	99.58±22.322	99.658±20.287	106.611±31.6	142.19±18.103***
**LV Vol;d (uL)**	62.089±10.473	72.612±26.612	108.153±25.423	87.932±18.277*	111.03±20.054	100.294±17.733	114.43±23.865	85.573±31.825**	137.656±30.451	106.166±23.24**
**LV Vol;s (uL)**	37.016±5.865	44.908±18.284	70.848±21.382	58.495±14.767	79.948±21.108	63.624±19.351*	80.747±26.068	50.57±27.7**	103.031±30.644	59.144±19.497***

Wild type C57BL/6 mice were treated with myocardium infarction (MI) surgery, immediately after the ligation of LAD coronary artery, AAV-LncHrt or AAV-CTRL in a total volume of 30 uL were injected into ventricle muscular wall but not ventricular cavity. Echocardiography analyses of cardiac function were performed at multiple time points post-MI surgery. N of each group was indicated. *: $P<0.05$; **: $P<0.01$ vs. control group. IVS;d: Interventricular septal thickness at diastole; IVS;s Interventricular septal thickness at systole; LVID;d: Left ventricular end diastolic internal dimension; LVID;s: Left ventricular end systolic internal dimension; LVPW;d: Left ventricular end diastolic posterior wall dimension; LVPW;s: Left ventricular end systolic posterior wall dimension; EF: Ejection fraction; FS: Fractional shortening; LV Vol;d: Left ventricular end diastolic volume. LV Vol;s: Left ventricular end systolic volume.

Supplementary Table 8. Echocardiography examination of cardiac function of AAV-LncHrt or AAV-CTRL intra-myocardial injected heart of sham operated heart.

	1 day post-MI		1 Week post-MI		2 Weeks post-MI		4 Weeks post-MI		6 Weeks post-MI	
	AAV-CTRL (n=6)	AAV-LncHrt (n=6)	AAV-CTRL (n=6)	AAV-CTRL (n=6)	AAV-CTRL (n=6)	AAV-LncHrt (n=6)	AAV-CTRL (n=6)	AAV-LncHrt (n=6)	AAV-CTRL (n=6)	AAV-LncHrt (n=6)
IVS;d (mm)	0.833±0.067	1.131±0.337	0.99±0.135	0.978±0.074	1.142±0.16	1.075±0.253	1.034±0.229	0.96±0.157	1.052±0.189	1.084±0.139
IVS;s (mm)	1.204±0.083	1.483±0.242	1.489±0.227	1.462±0.113	1.572±0.157	1.54±0.262	1.479±0.255	1.452±0.195	1.625±0.24	1.623±0.177
LVID;d (mm)	3.573±0.468	3.677±0.385	3.372±0.279	3.151±0.261	3.163±0.349	3.097±0.215	3.076±0.256	3.09±0.283	3.112±0.368	3.03±0.373
LVID;s (mm)	2.374±0.503	2.497±0.296	1.839±0.379	1.69±0.316	1.722±0.447	1.707±0.278	1.726±0.32	1.69±0.365	1.616±0.211	1.538±0.3
LVPW;d (mm)	0.671±0.072	0.744±0.123	0.715±0.091	0.695±0.069	0.957±0.227	0.915±0.321	0.681±0.073	0.878±0.212	0.802±0.157	0.888±0.106
LVPW;s (mm)	1.02±0.16	1.134±0.130	1.282±0.239	1.286±0.161	1.496±0.177	1.401±0.291	1.178±0.306	1.422±0.138	1.377±0.144	1.467±0.199
EF (%)	63.529±9.393	60.958±5.766	77.463±8.908	78.478±8.092	77.622±9.754	77.25±7.19	76.168±7.67	77.053±9,977	80.338±4.692	81.738±5.539
FS (%)	34.096±6.629	32.092±4.038	45.753±7.95	46.55±8.066	45.98±9.401	45.03±6.429	44.131±7.362	45.448±9.812	47.901±5.169	49.403±5.771
LV Mass (mg)	90.44±17.774	128.182±30.121	97.89±16.044	85.268±9.893	124.15±47.926	109.092±36.644	87.496±29.364	95.961±22.651	98.368±26.652	104.086±24.867
LV Mass (Corrected, mg)	72.352±14.219	102.545±24.097	78.312±12.835	68.215±7.914	99.32±38.341	87.274±29.315	69.997±23.491	76.769±18.121	78.694±21.322	83.269±19.894
LV Vol;d (uL)	54.641±16.4	58.081±14.361	46.906±9.849	39.817±8.209	40.467±10.815	38.074±6.30	37.567±7.838	38.044±8.794	38.996±9.769	36.609±10.882
LV Vol;s (uL)	20.907±10.069	22.695±6.497	10.964±5.857	8.724±3.669	9.623±6.508	8.846±3.85	9.203±4.242	8.881±4.667	7.555±2.407	6.88±3.658

Wild type C57BL/6 mice were sham operated without ligation of LAD coronary artery, AAV-LncHrt or AAV-CTRL in a total volume of 30 uL were intra-myocardial injected into ventricle muscular wall but not ventricular cavity. Echocardiography analyses of cardiac function were performed at multiple time points post-MI surgery. N of each group was indicated. *: P<0.05; **: P<0.01 vs. control group. IVS;d: Interventricular septal thickness at diastole; IVS;s Interventricular septal thickness at systole; LVID;d: Left ventricular end diastolic internal dimension; LVID;s: Left ventricular end systolic internal dimension; LVPW;d: Left ventricular end diastolic posterior wall dimension; LVPW;s: Left ventricular end systolic posterior wall dimension; EF: Ejection fraction; FS: Fractional shortening; LV Vol;d: Left ventricular end diastolic volume. LV Vol;s: Left ventricular end systolic volume.

Supplementary Table 9. Purine content of of AAV-LncHrt or AAV-CTRL intra-myocardial injected heart 6 weeks post MI detected by HPLC.

Sample	ATP Ret. Time	ATP area	ADP Ret. Time	ADP area	AMP Ret. Time	AMP area
CTRL-1	11.308	2511369	11.484	127430	11.751	266514
CTRL-2	11.285	3330839	11.474	118480	11.759	304141
CTRL-3	11.394	2062545	11.557	55851	11.794	207473
CTRL-4	11.386	4070872	11.556	141025	11.786	444288
CTRL-5	11.366	1938411	11.53	91662	11.82	218037
CTRL-6	11.378	4049342	11.532	109387	11.775	466673
CTRL-7	11.365	2144785	11.528	87629	11.778	210113
CTRL-8	11.393	1793194	11.557	50487	11.793	197203
LncHrt-1	11.272	5067515	11.464	62619	11.742	458219
LncHrt-2	11.352	3246329	11.514	49588	11.759	331756
LncHrt-3	11.316	3762797	11.482	69776	11.776	359967
LncHrt-4	11.382	2064607	11.546	41965	11.785	160734
LncHrt-5	11.38	2633993	11.546	58946	11.78	215467
LncHrt-6	11.39	2632932	11.554	43355	11.79	223609
LncHrt-7	11.317	1706846	11.493	72195	11.753	147394
LncHrt-8	11.32	4069185	11.489	70857	11.774	357258

Supplementary Table 10. QPCR primers used in this study.

Genes	Species	Purpose	Forward	Reverse
LncHrt	Mice	qPCR/RIP-exon1	TGGCACTAGCAAAAGGGTGATA	ACTCATCACTGGAGCCTACGA
	Mice	RIP qPCR-exon2	GGTTGAGTATTGGTAGTCATGTCAG	GAGACGGGATCTCACGATGT
	Mice	RT-PCR	ACATGGGACTCACAGCACTAAGTGCTTAGTG	GGAGACGGGATCTCACGATGTAGTGTTG
	Human	qPCR-exon1	TTGCGAATCTGTCACTTAAAATCCTAGGGT	GCATTCCTCAAAACAAGCTAAGCCATCTC
	Human	qPCR-exon2	GAATGGCTGACAGTCTTGGCTC	GGGAAATGACCAACAAGCAACT
NR045336	Mice	qPCR	TGTGTGGAGAGACAACGACC	CAAAACTGGCTCAGTGGGGT
XR373578	Mice	qPCR	GGTCATGCTCCTGTCGATGT	TGATGGCTGTGGTATGTGGG
NR131053	Mice	qPCR	CATTGCATCATCACAGCGGA	AAACACAGTGAGTGGCGTTC
XR386048	Mice	qPCR	CCAGGTTGGCTGAGATACCC	CAGTGCCTTTTGTCCACACC
Tnnt2	Mice	qPCR	CATCGACCACCTGAATGAAG	TTTCGCAGAACGTTGATTTC
Col3a1	Mice	qPCR	TCCCCTGGAATCTGTGAATC	TGAGTCGAATTGGGGAGAAT
U6	Mice	qPCR	GGAACGATACAGAGAAGATTAGC	TGGAACGCTTCACGAATTTGCG
Rn18S	Mice	qPCR	TCCGACCATAAACGATGCCG	CAATCTGTCAATCCTGTCCGTGTC
Actab	Mice	qPCR	GGAGCACCCTGTGCTGCTCA	GCCAGGTCCAGACGCAGGAT
Nppa	Mice	qPCR	CACAGATCTGATGGATTTCAAGA	CCTCATCTTCTACCGGCATC
Nppb	Mice	qPCR	GTCAGTCGTTTGGGCTGTAAC	AGACCCAGGCAGAGTCAGAA
Myh6	Mice	qPCR	GGG CTG GAG CAC TGA GAG	GAG AGA GGA ACA GGC AGG AA
Myh7	Mice	qPCR	CGCATCAAGGAGCTCACC	CTGCAGCCGCAGTAGGTT
	Human	qPCR	CCATCCCCACTTTGTACGTT	ACCAGGGGGTTGTCCATC
Fbp2	Mice	qPCR	GAAGAGAATAAAGAGGCGGTGA	AGGGTCAAAGCAAACCACAT
Ldhb	Mice	qPCR	GGCAGAGAAGCTTGGCATT	AGACTCCTGCCACATTCACC
Pkm	Mice	qPCR	GCAGGAACCGAAGTACGC	TGTGTTCCAGGAAGGTGTCA
Acaa2	Mice	qPCR	AAATGTGCGCTTCGGAAC	CGTTAATCCTGCCCACAAAG
Cpt2	Mice	qPCR	CCAAAGAAGCAGCGATGG	TAGAGCTCAGGCAGGGTGA
Acadl	Mice	qPCR	GCTTATGAATGTGTGCAACTCC	CCGAGCATCCACGTAAGC
Acsl1	Mice	qPCR	CCAAACCAGCCCTATGAGTG	CTTGAACCCCTTCTGGATCA
Acox1	Mice	qPCR	GCCCAACTGTGACTTCCATC	GCCAGGACTATCGCATGATT
Mdh1	Mice	qPCR	TGCTCTACTCATTCCCTGTCG	CCTTTGCTGTCAGGTCCATC
Pfkm	Mice	qPCR	GGACAATCTGCAAGAAAGCA	TGATGCTCTTCATGGGTCAT
Aco2	Mice	qPCR	CACAAAATGGCGCCTTACA	GACAGAGGCCACATGGTACTG
Cs	Mice	qPCR	GGAAGGCTAAGAACCCTTGG	TCATCTCCGTCATGCCATAGTA
Idh3g	Mice	qPCR	TCTCCTCTGCCGTCCTTG	TGTTGTGAGGAAATGCTCCTT
Idh3a	Mice	qPCR	CAGGTGACAAGAGGTTTTGCT	TGAAATTTCTGGGCCAATTC
Acss1	Mice	qPCR	CCACCAAGATCGCCAAGTA	ATCTGGTTTTGGGGAGACG
Hadha	Mice	qPCR	TTCTTAAAGACACCACAGTGACG	CTTCTTCACTTTGTCGTTCAGC
Hadhb	Mice	qPCR	GATGGAGGCCAGTATGCTTT	AGTCGGTCGCCTCCTTCTA
KLHL33	Mice	qPCR	AACTCAAGAGAGGGCAAGCAG	GCAACAGAACCACAAACGCC
Sirt2	Mice	qPCR	GTGCAGGAGGCTCAGGATTCAG	TCCTTCGAGGGTCAGCTCGT
Cdk5	Mice	qPCR	CCCAGCTACAACATCCTTGGT	AGTCAGAGAAGTAGGGGTGCT

Supplementary Table 11. Cloning primers used in this study.

LncHrt	Mice	AAV cloning	ACGACTCACTATAGGACTCACAGCACTAAGTG CTTAGTGAGGTTACTCTAGAG	GGGTCGACTCTAGAGGGAGACGGGATCTC ACGATGTAGTGTTG
	Mice	Lenti-virus-s1m cloning	CGCATCTGCTGGGCGACTCACAGCACTAAGT GCTTAGTGAGGTTACTCTAGAG	TCCTTCGCGGCCGCGGGAGACGGGATCTC ACGATGTAGTGTTG
	Mice	Lenti-virus cloning	ATTCTAGAGCTAGCGACTCACAGCACTAAGTG CTTAGTGAGGTTACTCTAGAG	TCCTTCGCGGCCGCGGGAGACGGGATCTC ACGATGTAGTGTTG
S1m	N.A	cloning	CTAGCGTAGAAAATGCGGCCGCCGACCAGAA TCATGCAAGTGCGTAAGATAGTCGCGGGTCG GCGGCCGCATCTGCTGGGG	AATTCCCCAGCAGATGCGGCCGCCGACCC GCGACTATCTTACGCACTTGCATGATTCTG GTCGGCGGCCGCATTTTCTACG

Supplementary Table 12. LncHrt RACE primers.

Primer name	Primer sequence
LncHrt-5' race	CAAGCTGTCCTCTGACTGCACATAAGCAC
LncHrt-3' race	GCAGGCGTTGACCTTACTGCTCTCTTG
LncHrt-5' race-nested	CAGCTTCCTGCTCTATGTTGTCTGAGGC
LncHrt-3' race-nested	GCTTGAGTGGAGCCATTGGACAGG

Supplementary Table 13. ShRNA and siRNA primer sequences.

Primer name	Anti-sense primer sequence
LncHrt-shRNA-A(AAV-LncHrt KD-A)	TTGTATAACTCCAATGCTCTT
LncHrt-shRNA-C (AAV-LncHrt KD)	TAGAAGATGAGACAGAGCTTT
Si-CDK5	UUGAGUAGACAGAUCUCCCTT

Supplementary Table 14. Antibodies used in this study

**Primary antibodies**				
**Antigen**	**Company**	**Catalog #**	**Origin**	**Working dilution**
HKI	HUABIO	ET1609-28	Rabbit	1:500 for WB
Cs	HUABIO	ET1706-40	Rabbit	1:500 for WB
PGK1	HUABIO	ET1609-63	Rabbit	1:500 for WB
IDH2	HUABIO	ET1704-93	Rabbit	1:500 for WB
PKM	HUABIO	ER1901-90	Rabbit	1:500 for WB
LKB1	HUABIO	ER1912-4	Rabbit	1:500 for WB
p-LKB1 (Ser428)	Bioss	bs-3249R	Rabbit	1:500 for WB
LKB1	Santa Cruz	SC-32245	mouse	6-8ug /IP
Acetylated Lysine	CST	9441	Rabbit	1:500
PDH	CST	3205	Rabbit	1:1000 for WB
p-AMPKα(Thr172)	CST	2535	Rabbit	1:1000 for WB
AMPKα	CST	5831	Rabbit	1:1000 for WB
CDK5	CST	14145	Rabbit	1:1000 for WB, 1:50 for IP
GAPDH	CST	5174	Rabbit	1:5000 for WB
Actin	CST	4970	Rabbit	1:5000 for WB
IgG	CST	2729	Rabbit	2~5 ug/IP
SIRT2	Abcam	ab211033	Rabbit	1:2000 for WB, 1:30 for IP
